# A systematic review of interventions to support adults with ADHD at work—Implications from the paucity of context-specific research for theory and practice

**DOI:** 10.3389/fpsyg.2022.893469

**Published:** 2022-08-22

**Authors:** Kirsty Lauder, Almuth McDowall, Harriet R. Tenenbaum

**Affiliations:** ^1^Centre for Neurodiversity Research at Work, Birkbeck College, London, United Kingdom; ^2^School of Psychology, University of Surrey, Surrey, United Kingdom

**Keywords:** attention deficit hyperactivity disorder, ADHD, workplace, systematic review, interventions, treatment, work

## Abstract

Attention Deficit Hyperactivity Disorder (ADHD) is estimated to affect 3.5% of the global workforce. Despite the high prevalence rate, little is known about how best to support adults with ADHD (ADHDers) at work. Relevant research is dispersed across different disciplines such as medicine, health studies and psychology. Therefore, it is important to synthesize interventions aimed at ADHDers to examine what learning can be gleaned for effective workplace support. We conducted a systematic review of relevant interventions framed by realist evaluation and the Context-Intervention-Mechanism-Outcome classification to identify key mechanisms of effectiveness for workplace interventions. We searched 10 databases including a range of journals from medical science to business management applying predetermined inclusion criteria and quality appraisal through a risk of bias assessment for quantitative and qualitative methods. We synthesized 143 studies with realist evaluation. Most studies evaluated the effectiveness of pharmacological interventions highlighting the dominance of the medical approach to supporting ADHDers. Key mechanisms of effectiveness were identified from psychosocial interventions including group therapy, involvement of people in the ADHDers network, and the importance of the client-patient relationship. Overall, there is limited research that examines the effectiveness of workplace interventions for ADHDers. Furthermore, much of the existing research evaluates pharmacological interventions which is difficult to transfer to the workplace context. It is recommended that future research and practice consider the key mechanisms identified in this review when designing interventions as well as barriers to accessing support such as disclosure and self-awareness.

## Introduction

Attention deficit hyperactivity disorder (ADHD) is a multidimensional neurodevelopmental condition that has only recently been considered to impact the lifespan (Caye et al., 2016). In the UK, ADHD is formally diagnosed by a psychiatrist where marker symptoms are inattention and hyperactivity/impulsivity (Tatlow-Golden et al., 2016) in varied constellations. Other symptoms experienced are difficulties with emotional regulation and challenges with social interactions (Pitts et al., 2015; Corbisiero et al., 2017). The conceptualization of ADHD is debated and often pathologized in line with the core symptoms. However, recent conceptualisations consider ADHD to be part of neurodiversity and conceptualized through a biopsychosocial model shifting the focus to understanding difference (Doyle, 2020) rather than framing ADHD as a burden (Asherson et al., 2012). Regardless of conceptualization of ADHD, the range of symptoms associated with the condition impacts on all functional domains from personal life to the workplace; for a formal diagnosis these need to be present in more than one life domain (Davidson, 2008; American Psychiatric Association, 2013). The estimated 3.5% of the global workforce who are likely to have ADHD (de Graaf et al., 2008) are likely to report issues with work performance, difficulties in job retention, under- and unemployment, and negative work-related well-being (Küpper et al., 2012; Adamou et al., 2013; Painter et al., 2017). Therefore, it is imperative that adequate workplace support is in place to mitigate such likely negative outcomes (Adamou et al., 2013).

The National Institute for Health Care Excellence (2019) guidelines that clinicians apply when recommending how to manage ADHD state that medications (referred to in the present review as pharmacological interventions) are the first line of treatment once environmental modifications, through reasonable adjustments, have been implemented and reviewed. Non-pharmacological (also referred to as psychosocial) interventions are only recommended if the ADHDer does not want to use medication, has difficulty adhering to medication, or found it ineffective (National Institute for Health Care Excellence, 2019). Gaining and sustaining good work is imperative for well-being for everyone, including neurominority populations. However, little is known about what workplace environmental modifications are effective and whether pharmacological or psychosocial interventions are at all effective in workplace contexts to enhance work outcomes, including individual performance. Thus far, no comprehensive review of interventions for adult ADHD has focused on work-related studies or scrutinized the organizational/management literature (De Crescenzo et al., 2017; Fullen et al., 2020). Given habitual challenges experienced by ADHDers regarding effective functioning and thriving careers, such as concentration challenges being interpreted as a sign of underperformance, rather than supported through adjustments, it is important to revisit the existing literature to identify studies that examine relevant interventions to recognize any effective mechanisms transferable to a work context. Somewhat contrary to the broad NICE guidance, existing reviews have suggested psychosocial interventions as more effective than pharmacological when addressing functional outcomes such as quality of life or co-occurring challenges associated with ADHD such as anxiety or depression (Linderkamp and Lauth, 2011; Lopez et al., 2018), and thus may be more pertinent and appropriate in a work context.

### Objectives

Using realist synthesis and evaluation, the present review aims to synthesize, (a) the respective types of support/ interventions available to adult ADHDers and (b) the evidence for their effectiveness in workplace contexts or on any work-relevant outcomes.

### Review approach

As it was our aim to take a cross disciplinary review including organizational and management literature as well as complex interventions derived from clinical and health context, we took a realist evaluative approach (Pawson and Tilley, 1997). With foundations in programme theory (identifying the underlying theory about why an intervention is effective), realist evaluation emphasizes the importance of context where researchers are advised to focus on “what works for whom, in what circumstances, in what respects and over which duration” (p. 15) rather than surmising whether the type of intervention is effective or not (Pawson, 2013). Drawing on the premise that underlying theory provides the answers to why and how interventions work in some circumstances, but not in others (Astbury and Leeuw, 2010) we applied the CIMO-logic (Denyer et al., 2008). The context (C) is defined as the environment or human factors, the intervention (I) as specified in the research question, the mechanisms (M) created by the intervention as the key components for its efficacy, and the outcomes (O) ranging from performance to cost reduction (Denyer et al., 2008) also encompassing potential interactions between respective units of analysis (for an example see Doyle and McDowall, 2019) throughout the review process including the review question, data extraction, quality assessment, and interpretation of findings.

## Methods

### Expert panel, review process and review question

**Figure 1:**
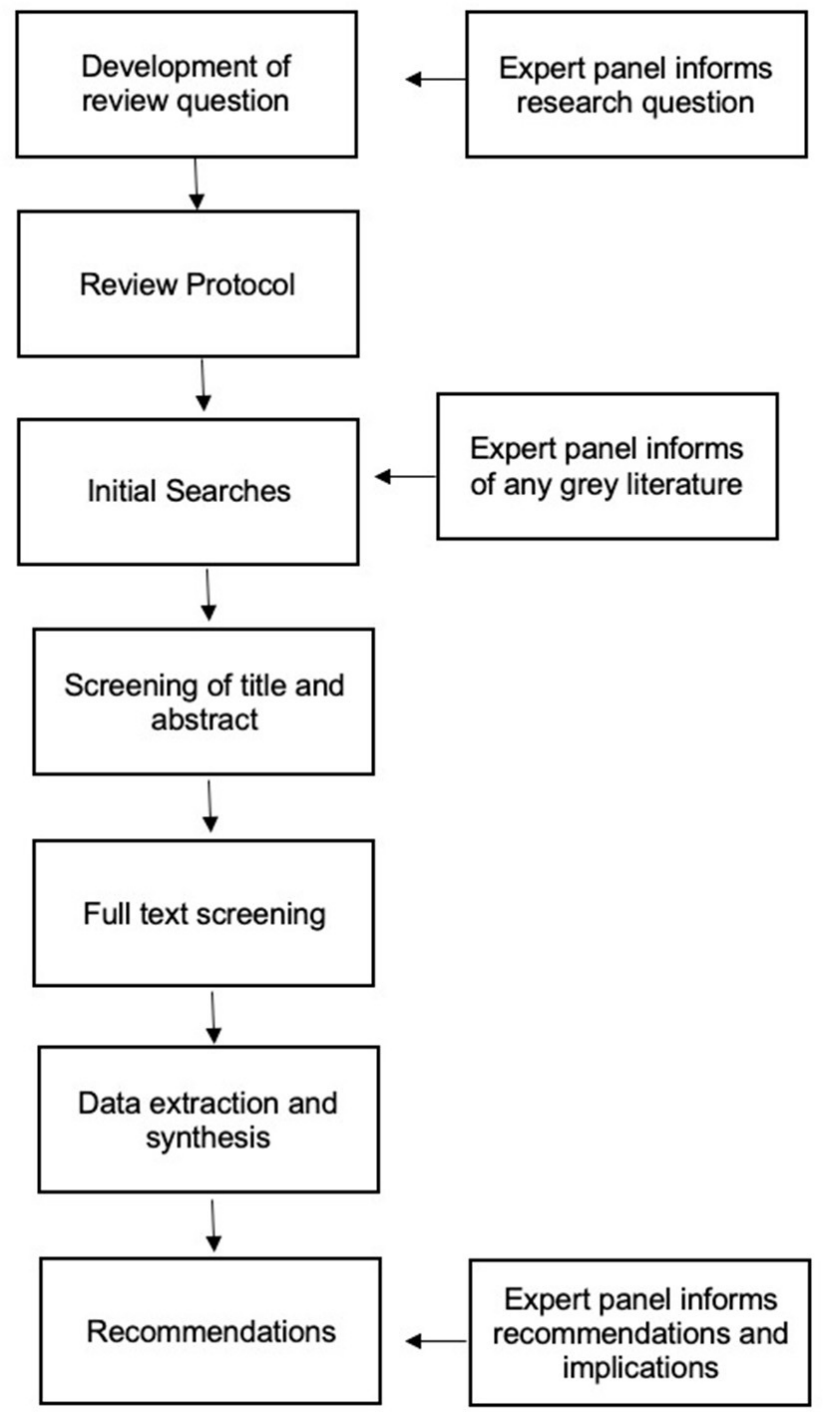
Systematic review method.

In line with best practice guidelines and prior research (Gough et al., 2012; Beauséjour et al., 2013), the current review incorporated an expert panel consultation (*N* = 4) at multiple points in the review process (see Figure 1). The panel included practitioners working in employment support for ADHDers, an academic whose research focuses on support for ADHDers, and a psychoanalyst who works therapeutically with ADHDers. Two members of the expert panel disclosed that they had received a diagnosis of ADHD and hence took the dual role of potential ‘service users' as recommended in reviews where the public may be impacted by the findings (Gough et al., 2012).

The experts were invited to answer nine review scoping questions including their views on definitions for ADHD, the most effective intervention for ADHDers is and comment on and if necessary suggest amends for the protocol review questions. Their responses were analyzed using content analysis to identify common themes as summarized in Table 1 (Krippendorff, 2004). Adult ADHD was conceptualized by the panel to encompass multiple domains and contexts which affect the life span. The panel stressed difficulties beyond the core symptoms such as working memory, self-regulation, and the high prevalence of co-occurrences. Strengths were also highlighted in some panel members' answers including enthusiasm, passion, and loyalty. Regarding the most effective intervention, psychoeducation and coaching were the most common response, emphasizing that these required delivery from a trained specialist working with ADHDers. The experts identified broad research gaps including interventions such as diet and exercise, managing specific behaviors and relationships, and the impact of diagnosis and stigma on the individual. Finally, workplace related research was identified as a priority for academia in the next 5 years given the paucity of primary evidence.

**Table 1:** Findings from the expert panel at the research question stage.

**Definition of adult ADHD**	**Effective interventions**	**Efficacy of psychological interventions**	**Research gaps**
• Lifelong • Working memory • Concentration • Strengths- enthusiasm, passion, loyalty, novelty. • Invisible disability • Self-regulation • Affects multiple domains/ cross-contextual • Co-morbid	• Coaching- the coach must be experienced with ADHD • Technology • Exercise • Medication, initially rather than long-term and • Psychoeducation • Interventions involving the support network around the person • Separate treatment for co-morbidities	• Coaching is effective in boosting work-related performance • Group sessions	• Diet and exercise • Managing hoarding and compulsive behaviors • Improving awareness among public-body decision-makers and GPs • ADHD presentation in females • ADHD in relationships • Workplace support • Stigma and marginalization
	• Targeted at organizational challenges- developing strategies in a job that matches interests		• Success narratives • Impact of diagnosis on career success guidelines

#### Review questions

The panel agreed our review questions and scope; following some discussion we agreed to keep the focus broad and international notwithstanding differences in legislative frameworks which may impact on how any interventions are delivered. Guided by the CIMO framework, the final overarching review questions were: *Which interventions, documented in the literature, aim to support adult ADHDers?*

(a) *In which contexts have any studies been conducted*,
(b) *How can we classify types of intervention*,
(c) *What are the mechanisms in the interventions, and*
(d) *What are the outcomes addressed?*

### Study design

Our inclusion criteria stipulated that participants in primary studies had to be over the age of 18 years and received a formal ADHD diagnosis using the DSM 3, 4, or 5 criteria in the treatment/intervention group prior or at the beginning of the intervention from a clinical practitioner. The intervention had to meet the following definition: “…activities, techniques, or strategies that target biological, behavioral, cognitive, emotional, interpersonal, social, or environmental factors with the aim of improving health functioning and well-being” (Institute of Medicine of the National Academies, 2015, p. 31).

Furthermore, the interventions had to be preventative or therapeutic and not be purely diagnostic or prognostic (Santos et al., 2007) including combinations of pharmacological and psychological treatments. Interventions based on altered brain stimulation were excluded because (1) they tend to have a diagnostic purpose, and (2) they are not currently recommended for supporting adult ADHD (Kooij et al., 2010; National Institute for Health Care Excellence, 2019). We excluded pilots, protocols, systematic reviews and meta-analyses because of our focus on specific primary interventions. The outcomes or findings from the study had to be measurable and defined as an “expected result” consistent with the PICO framework (Santos et al., 2007, p. 510). We excluded interventions that did not assess the “expected result” was excluded, for example, interventions solely assessing adverse effects of the drug treatments as well as studies solely assessing outcomes non-transferable to the workplace such as physiological changes. Qualitative primary studies were included where relevant as they are suited to eliciting process focused evidence (“how”). Finally, no date restriction was placed on the searches, studies could be published or unpublished but had to be written or translated into the English language.

### Systematic review protocol

Our review protocol was registered with PROSPERO, an international register of prospective systematic reviews (registration number CRD42018092237).

### Search strategy

We searched databases from a variety of disciplines, including organizational and management journals and those specific to ADHD as shown in Table 2. Our search terms were agreed by dividing the research question into its individual elements with consultation from a specialist subject librarian and are shown in Table 3 (Petticrew and Roberts, 2006).

**Table 2:** Databases.

**Medical, science, psychology and business databases**	**ADHD specific journals**
Academic search complete	ADHD Attention Deficit and Hyperactivity Disorders
Business source premier	Journal of Attention Disorders
Criminal justice abstracts with full text	
Library, information science and technology abstracts	
PsycARTICLES	
PsycINFO	
MEDLINE	
ProQuest Business collection	
Scopus	
Web of science	

**Table 3:** Search terms.

**Element**	**Variations**
Adult ADHD	Adult ADHD, Adult ADD, Adult Attention Deficit Hyperactivity Disorder, Adult Attention Deficit Disorder, adults with ADHD, adults with attention deficit hyperactivity disorder, adults with ADD, adults with attention deficit disorder
Interventions	Intervent*, treat*, manag*, program*, counsel*, coach*, therapy, trial, train*

^*^represents the wildcard in searches.

### Data extraction

We undertook an inductive open-coding approach during the data extraction (Oliver and Sutcliffe, 2012) including some predetermined categories such as study design and participant gender ratios. During extraction we identified additional categories including the intention-to-treat analysis (defined as a type of analysis that includes data from participants who have dropped out in the latter stages) and placebo-controlled interventions. We developed further criteria for extracting information about study quality including ratings according to whether primary studies had answered their research question (Jarde et al., 2012).

## Results

### Study selection and characteristics

**Figure 2:**
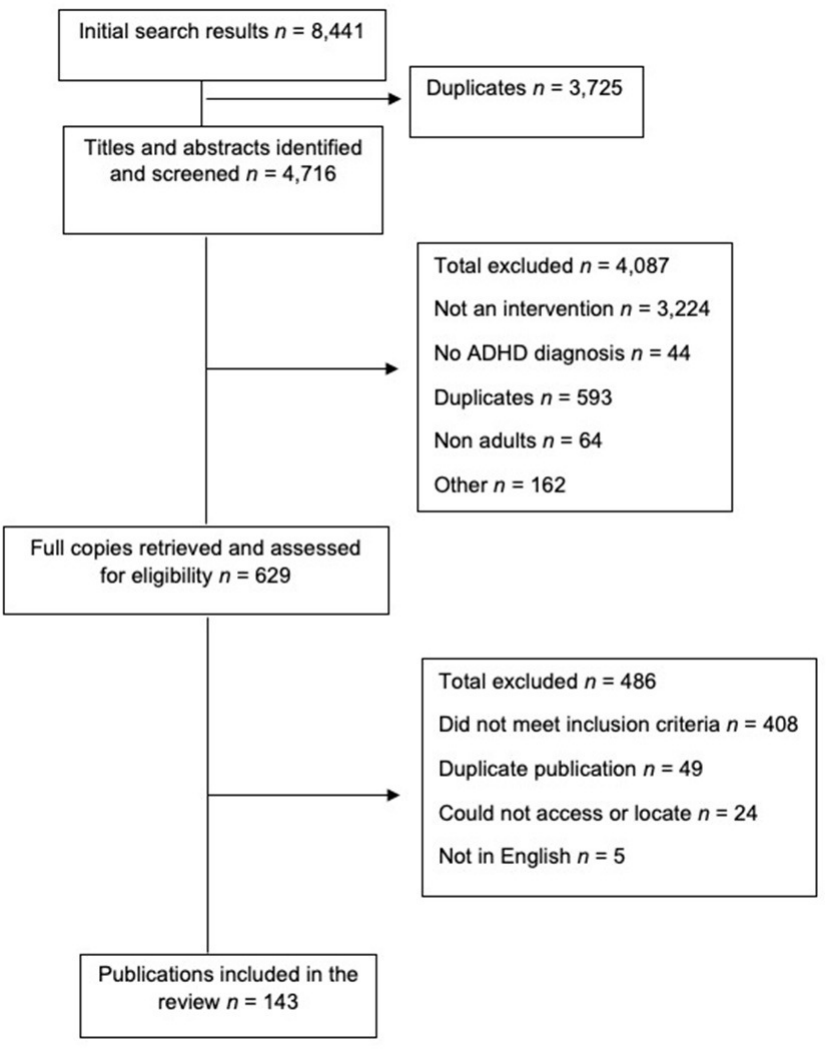
Flow chart of the reviews screening process using the PRISMA guidelines.

EPPI Reviewer was used to manage the data and record the decision making (Thomas et al., 2020). The first step involved screening the study titles and removing any duplicates. The lead author screened the title and abstracts against the inclusion criteria. A member of the review team then screened 5% of the title and abstracts and any disagreements were discussed and resolved. Following a percentage agreement of 97%, Cohen's kappa was calculated across the two reviewers and they had a score of κ = 0.86 indicating strong agreement (McHugh, 2012). The full text versions were retrieved and screened accordingly. If the full texts were not available, the reviewer emailed the author to request a copy. Figure 2 displays the PRISMA figure of the screening and selection process (Page et al., 2021).

### Findings

Our findings are presented in two parts. Part one provides a systematic map of the studies with their representative characteristics to give an overview of the interventions documented in the literature and the field in line with the aim of the review and the overarching review question. The second part contains a realist evaluative synthesis of the interventions discussing the contexts in which they have been studied, the mechanisms in the interventions, and the outcomes addressed.

#### Systematic mapping

**Figure 3:**
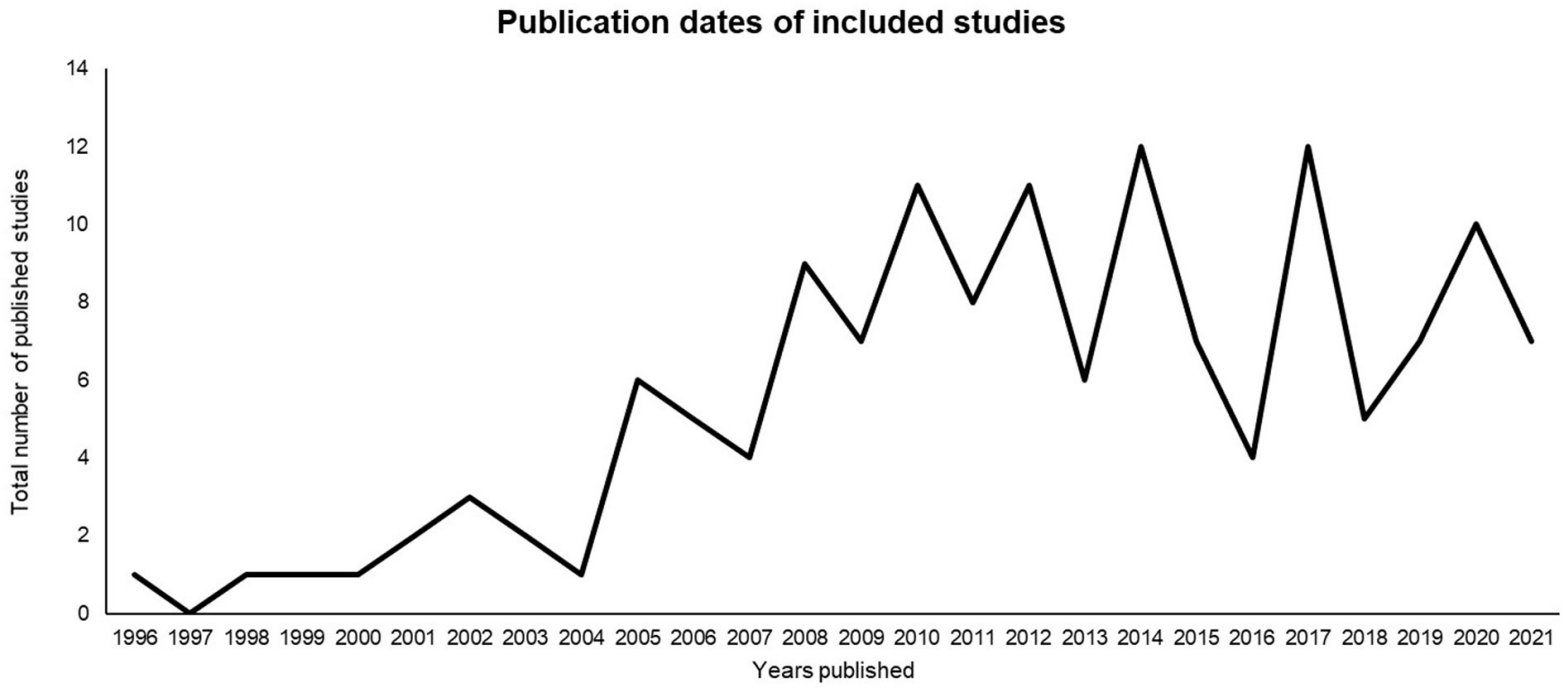
Publication dates of included studies.

We synthesized 143 articles. Each study is listed in Table 4 (Supplementary material) with its representative (a) year of publication, (b) intervention type, (c) country, (d) total sample, (e) gender ratio, (f) design, and (g) length of follow-up in weeks. The studies were published from 1996 to 2021 as presented in Figure 3. There are two peaks in publications that reflect prior systematic review findings and represent the contextual shifts in understanding and diagnosis of adult ADHD. In 2008, the National Institute of Clinical Excellence (NICE) guidelines were released which unlike previous versions, included methods to support adult ADHDers. The second and most significant peak in publications is after 2013 where the upgraded criteria for ADHDers was published in the Diagnostic Statistical Manual (DSM) version five (2013), which was the first time ADHD symptoms and experiences in adults were noted as part of the diagnostic criteria. Then a third peak occurred in 2019, when the NICE guidelines were updated to highlight the importance of environmental modifications and pharmacological intervention rather than previously combined pharmacological and psychosocial interventions.

A total of 22,132 participants were involved in the studies. The mean sample per study was 155. Studies had typically high levels of drop-out rates at follow up, ranging from 0 to 90% (Johnson et al., 2010). Follow-up length varied greatly, ranging from the same day to 4 years. The mean follow-up length was 16 weeks. Follow-up length was further classified into long-term and short-term with long term being 6 months or more (23% of studies) and short term <6 months (all other studies).

From the 143 studies, 60.8% were evaluating the efficacy of pharmacological interventions, 28.7% were classified as psychosocial interventions and the remaining 10.5% evaluated the efficacy of pharmacological combined with psychosocial interventions. We categorized study designs, similarly to a prior systematic review (Bower et al., 2001), into randomized control trials (RCT; *k* = 101), controlled before and after (CBA; includes control group; *k* = 14), and simple before and after (SBA; no control group; *k* = 28).

#### Realist synthesis

##### How can we classify types of intervention?

We classified interventions broadly into three groups depending on the underpinning theories and disciplines in which they were developed. The understanding that ADHD is a neurological imbalance in the brain is dominant in medical disciplines that argue the imbalance can be targeted by specific drugs and forms the first classification of *pharmacological interventions* (Durston, 2003). We based the second classification, *psychosocial* interventions, on theorisations that ADHD can be treated through psychological support (Young and Amarasinghe, 2010), and the final classification was entitled *combination interventions* which included studies where a multidisciplinary approach combining both pharmacological and psychosocial interventions is deemed most effective. Table 4 outlines our classifications and their representative underpinning theory and offers a breakdown of the sub-classifications of the interventions.

**Table 4:** Study classifications, sub-classifications, and their representative number of studies and underpinning theory.

**Main classification**	**Sub-classification**	**Number of studies**	**Underpinning theory (mechanisms)**
Pharmacological	Stimulants	48	Chemical imbalance in neural networks
	Non-stimulants	27	
	Anti-depressants	8	
	Mixture	4	
Psychosocial	Cognitive behavioral therapy	14	Impact of thought on behavior and emotions
	Skills training/Coaching	11	Psychoeducation
	Attention/cognitive training	8	Regulating attention and improving cognition
	Mindfulness	6	Regulating attention
	Alternate therapies	2	
Combination	Stimulant and psychosocial	15	Holistic approach

Most studies evaluated pharmacological interventions, assessing the efficacy of the three common drug treatments used to treat adult ADHD; Methylphenidate (MPH), Atomoxetine (ATX) and Lisdexamfetamine dimesylate (LDX) (*k* = 87). Pharmacological interventions can be further categorized according to their drug type as stimulants or anti-depressants. Caye et al. (2018) explain that psychostimulants such as Methylphenidate and Amphetamines are the first line of treatment for ADHD because they are the most researched. Second line treatments involve Atomoxetine and anti-depressants that are often prescribed if psychostimulants are contraindicated or not tolerated or when co-occurrence is present, especially in cases of ADHD and Bipolar Disorder, any substance abuse or Tourette's Syndrome (Caye et al., 2018).

A wide range of psychosocial interventions provided the basis for over a quarter (29%) of the studies which we further classified according to the theories forming the basis of the therapies including cognitive behavioral therapy, mindfulness, attention/cognition training, skills training and/or coaching, and alternative therapies.

The remaining 15 studies combined pharmacological and psychosocial interventions. Most of these studies were stimulant treatment combined with cognitive behavioral therapy (*k* = 9). Other psychosocial therapies combined with medication were mindfulness based cognitive therapy, group psychotherapy, and problem-focused therapy. One study advanced on the traditional two group comparison by comparing individual counseling to group psychotherapy in pharmacologically treated participants aiming to explore the most effective psychosocial treatment (Philipsen et al., 2007).

##### The group

We classified the interventions classified by mode of delivery. Of the 143 studies synthesized, 21 were delivered to a group or involved a combination of group and individual delivery. All 21 studies were classified as psychosocial or a combination of pharmacological and psychosocial. Of the 21 studies, 20 were classified as effective, 12 were conducted in European countries with 12 being conducted in universities, research centers or university hospitals. Group interventions are argued to be beneficial for sharing lived experiences, coping strategies, increasing feelings of belongingness and knowledge about ADHD (Bramham et al., 2009; Jackson et al., 2014; Fullen et al., 2020). Learning in a group also increases self-efficacy through higher levels of hope and motivation (Bramham et al., 2009; Tian et al., 2018). However, only six studies directly assessed outcomes relating to self-esteem or efficacy with one study additionally measuring social functioning. Therefore, it is difficult to compare the effectiveness of group vs. individual interventions because the theorized benefits are broad and not consistently measured.

##### Involvement of others

Group interventions tended to include individuals from the ADHDers support network as part of the intervention. Including significant others during the intervention is arguably effective and linked to psychoeducation whereby the ADHDer and their partners or family is empowered with knowledge about ADHD and how to better support it (Lukens and McFarlane, 2004). So far, research examining the efficacy of including family members in interventions for ADHD has focused on children and adolescents (Kaslow et al., 2012). Only five interventions in the present review involved someone in the delivery of the intervention that was not the clinician or individual with ADHD.

Of the five studies, four included the ADHDer's significant other or family member (Virta et al., 2008; Hirvikoski et al., 2011, 2017; LaLonde et al., 2013). The final study included a “support person” or “coach” for help with organizational tasks, and if the ADHDer did not have a support person from their own social network, an undergraduate student was allocated to them to adopt the support person role (Stevenson et al., 2002). All studies reported positive findings with improvements in outcomes beyond reducing symptoms like employment, maintaining relationships, organization skills, self-esteem, and knowledge about ADHD. In short, involving the support network around the ADHDer has marked effects on all outcomes emphasizing that an encouraging and supportive environment can increase the impact of an intervention.

##### In which contexts have the studies been conducted?

Contexts are defined as environmental factors that affect behavior change (Denyer et al., 2008). These contextual factors can be separated into four layers: the infrastructural system, the institutional setting, interpersonal relationships and the individual themselves (Pawson and Tilley, 1997). We mapped the 143 included studies onto the four areas with referrals being infrastructural system, location as an institutional setting, clinician-patient relationship as part of the interpersonal relationships and lastly, co-occurring at the individual layer.

##### Referrals and dropout

From the 143 studies 90 were outpatient referrals which means that the adults had received a diagnosis and were immediately referred by the psychiatrist for their first set of treatment at a specialist center or clinic (Kooij et al., 2010). Another method of recruitment was to advertise the intervention and remunerate participants with a formal diagnosis (*k* = 6). These two methods of recruitment attract and include participants who are already aware that they may have ADHD or have recently been diagnosed. Pawson (2013) suggests that with medication it is difficult to pinpoint the exact moment in which the intervention began, which is particularly true with outpatient referrals as basic knowledge or understanding of ADHD may exist prior to the referral for treatment. As a result of going through the diagnostic process, some level of psychoeducation, researching and learning about ADHD, might have already happened prior to medical treatment. Consequently, it is unclear whether the intervention began at the point in which the ADHDer began to learn about ADHD or at the point medication is initiated. This level of self-awareness and knowledge is difficult to measure and is likely to differ greatly between individuals. With an increase in easily available information like self-help and guidance online, it is important to consider how potential misinformation or accurate information may influence how different interventions are perceived before interventions begin.

Dropout rates varied greatly across the studies. Adherence is a central part of assessing the efficacy of an intervention (Horwitz and Horwitz, 1993). In pharmacological studies, adherence is measured through self-report during follow up sessions recording whether the participant has taken the medication or not. In psychosocial interventions, it is typically assessed by attendance. A review of medical adherence in children and adult ADHDers found non-adherence rates that ranged from 13.2 to 64% but concluded that there is minimal research addressing reasons for non-adherence in adults (Adler and Nierenberg, 2015). Qualitative research has identified forgetfulness, a challenge for ADHDers, and lack of guidance from clinicians as potential barriers to medication treatment adherence (Matheson et al., 2013). Therefore, it important for future research and practice to consider adherence as an influential factor in the efficacy of pharmacological interventions and identify and remove any potential barriers.

##### Location

The most common settings for pharmacological studies were outpatient clinics or multi-center clinics in North America (*k* = 47). The majority of participants had been referred to the clinic, received a formal diagnosis and then received a treatment. Assessing the effectiveness of pharmaceutical interventions across multiple clinics not only provides researchers and clinicians with information about the impact of the drug in multiple countries, but also provides an indication of the prevalence of ADHD across cultures (Polanczyk et al., 2007; Wang et al., 2017). Research centers and university settings (*k* = 24) were more likely to study the effectiveness of psychosocial interventions, but unlike common critiques of a student sample and the lack of ecological validity, only two of these studies recruited student samples indicating good generalisability (Ward, 1993).

##### The clinician-patient relationship

Prior research has drawn attention to the significance of the patient and clinician relationship as well as healthcare outcomes (Kelley et al., 2014). As a potential mechanism, it is argued that the better the quality of the relationship, the quicker the recovery and the higher the rate of adherence (Thompson and Mccabe, 2012). The relationship between the clinician-patient is often layered and dynamic making it difficult to directly assess or compare between studies (Street et al., 2009). Key components of an effective relationship are similar in medical and educational settings with research suggesting the following: good management of emotion, high patient or coachee knowledge of their own condition, client/coachee centered approaches and good communication with shared understanding (Street et al., 2009; Jackson et al., 2014; Kelley et al., 2014; Lai and McDowall, 2014). Many of the 143 included studies relied on clinician ratings of symptoms and rarely included the patient. In addition, nearly all the studies had no means of measuring the impact of the clinician/patient relationship with two measuring patient experience directly. In context, as many of the studies were in outpatient clinics, the initial appointment for the treatment was most likely the first point of contact after receiving a diagnosis for a large proportion of the ADHDers, enhancing the importance of a positive and meaningful interaction. Although no measures or understandings were assessed in these studies. We therefore identify the clinician-patient relationship as potentially influential and recommend further investigation.

##### Co-occurrence

As a diagnosis, ADHD is rarely present without co-occurrences, clinicians suggest that co-occurrence with depression and anxiety is particularly prevalent due to the experiences of failure, lack of support, and challenges with regulating emotion (Jensen et al., 1997). In experimental study designs, co-occurrence is considered a confounding variable because it is difficult to isolate any beneficial effects of an intervention to a specific condition (Fortin et al., 2006). Consequently, most studies excluded co-occurrences as part of their criteria (*k* = 135), whereas others deliberately addressed ADHD with co-occurrences like social anxiety disorder or substance use disorder (*k* = 8). In contrast, there is an argument as to whether removing or excluding participants with co-occurring conditions lessens the external validity because they provide an unrealistic view of ADHD (Fortin et al., 2006).

##### What are the mechanisms in the interventions?

Mechanisms can be defined as the processes or underpinning methods in which the intervention operates in a specific context to produce a specific outcome that can be triggered in some contexts and not in others (Denyer et al., 2008; Dalkin et al., 2015). We understand mechanisms as the fundamental processes in which interventions are expected to be effective, relating them to the disciplines in which the interventions were developed and theorized, for example, brain chemistry, cognition, and psychoeducation (Denyer et al., 2008).

##### Brain chemistry

A large proportion of the studies were pharmacological in nature. From our realist synthesis and evidence-based medicine perspective, randomized controlled drug interventions are the gold standard because the mechanisms, contexts and outcomes have been explored years before the randomized control trial is carried out (Pawson, 2018). For interventions involving stimulants, the process prior to testing the stimulant in humans is extensive (Lipsky and Sharp, 2001). Therefore, the theories and mechanisms have been well-established prior to the intervention.

##### Cognition

Cognitive models of ADHD explain a deficit in the prefrontal cortex which is responsible for executive function (Willcutt et al., 2005). Both Barkley (1997) and Brown (2006), leading researchers in ADHD, have developed theoretical models that explain ADHD as a difficulty in managing executive function resulting in impulsive and inattentive behavior. These models have been the foundations behind interventions such as cognitive behavioral therapy (CBT), cognitive remediation, and working memory or attention training. CBT was developed to address anxiety and depression by altering thought processes and behavior to avoid the repetitive negative thinking and corresponding behavior (Beck and Beck, 2011). In a Cochrane review, CBT is argued to treat ADHD by tapping into the negative thinking which has been a result of the negative experiences associated with the core symptoms (Lopez et al., 2018). ADHD is also highly co-occurring with anxiety and depression supporting the use of CBT to target co-occurring symptoms. The techniques used in CBT often include psychoeducation followed by the acquisition of techniques to address the individual challenges the person experiences (Huppert, 2009). Goal setting is an integral part of CBT and is useful in assessing effectiveness (Beck and Beck, 2011). Of the 23 studies that assessed the efficacy of CBT alone or with medication, eight were long term and half included measures of depression and/or anxiety finding positive effects in all studies (*k* = 11).

Mindfulness formed the intervention in seven of the studies and in many was combined with CBT (Bueno et al., 2015; Edel et al., 2017; Bachmann et al., 2018; Gu et al., 2018; Hoxhaj et al., 2018; Hepark et al., 2019; Nicastro et al., 2021). Similar to CBT, mindfulness aims to tap into cognition. However, CBT is focused on attention rather than other symptoms and aims to focus attention on the present, inner emotions and acceptance (Pirson, 2014). Most of these studies had positive effects on reducing symptoms, one found no difference in measures of memory (Bueno et al., 2015) and another found that mindfulness was effective for all participants, not just those with ADHD (Bachmann et al., 2018).

##### Psychoeducation

Psychoeducation aims to enhance a person's understanding of mental health by increasing the individual's knowledge and awareness of their condition and supporting them in sharing their experiences (Getachew et al., 2009). It also offers the opportunity for those supporting the individual such as family members, to help support ADHDers (Anderson et al., 1980). The idea is that self-awareness and knowledge is key in learning strategies to manage any condition or increase functioning rather than simply reduce the symptoms (Lotfi et al., 2010). In some areas, psychoeducation is argued to adopt a strengths-based approach (Lukens and McFarlane, 2004). Despite the strong evidence base as an intervention for affective disorders and preliminary evidence in interventions for children and adolescents with ADHD, there is limited research applying psychoeducation with ADHDers (Lukens and McFarlane, 2004; Dahl et al., 2020). Seven studies mentioned their use of psychoeducation typically combining it with another therapy or training (Wiggins et al., 1999; Hirvikoski et al., 2017; In de Braek et al., 2017; Bachmann et al., 2018; Hoxhaj et al., 2018; Gaur and Pallanti, 2020; Hartung et al., 2020). These studies indicated improvement in a range of outcomes including executive functioning, time management/organization skills, general functioning, and knowledge, as well as knowledge and coping in significant others. Therefore, we consider psychoeducation as a key mechanism that is worth incorporating in future design of interventions because of the variety of benefits for the ADHDer as well as significant others.

##### What outcomes have been addressed?

The outcomes assessed in each intervention varied greatly across the 143 studies. Initially, we classified the primary outcomes according to whether they involved a measurement of the core symptoms. Those outcomes beyond the core symptoms were further classified into what they assessed: behavior, cognition, physical/functioning, social, and person/emotion. We additionally discuss the outcomes in relation to short-term and long-term and qualitative methods.

##### Reducing core symptoms

Core outcome measures were defined as those assessing the three core symptoms of adult ADHD; impulsivity, inattention, and hyperactivity. Of the 143 studies, 108 assessed a reduction in core symptoms as their primary outcome. There are several validated measures for adult ADHD and in most of the studies a mixture of these measures was used pre and post intervention. The most popular being Conner's Adult ADHD Rating Scales (CAARS) (*k* = 44) and the ADHD Rating Scale (ADHD-RS) (*k* = 38). The authors of the ADHD-RS are prominent authors in the intervention studies and the CAARS was used to validate the measure. It is important to consider the authors as potential sources of bias because of their involvement in developing the measures.

##### Beyond the core symptoms

We categorized a range of outcomes beyond core symptoms further which are displayed with relevant examples in Table 5 alongside the number of studies involving these types of measures. The vast range in outcomes suggests the impact of ADHD to all aspects of life beyond the core symptoms, from social relationships to general self-esteem. Regarding effectiveness, primary outcomes related to cognition were associated with mixed or unclear results. Outcomes relating to social and emotion/person were assessed more often in psychosocial interventions with overall positive effects of the intervention. Aside from cognitive assessments of outcomes which typically involve using technology, and the Clinical Global Impression scale (*k* = 41), which is purely based on the clinicians rating, many outcomes were assessed using self-report rating scales. In one study, a strength of ADHD, creativity, was measured and improved after the administration of a stimulant which is in line with the expert panel, that strengths are important to consider (Kahn, 2008). Evaluating outcomes associated with strengths supports the neurodiversity conceptualization of ADHD and challenges the pathological approach.

**Table 5:** Outcomes by category with examples and total number of studies assessing them.

**Outcomes**	**Example scales**	**No of studies assessing outcomes**
Behavioral	On Time Management Organization and Planning scale (ON-TOP) Substance/Alcohol use	24
Cognitive	Continuous Performance Test (CPT) Verbal memory (WMS-R)	38
Physical/Functioning	Clinical Global Impression (CGI) Adult ADHD Quality of Life Scale (AAQoL) Global Assessment of Functioning (GAF)	76
Social	Social Adjustment Scale Self-Report (SAS-SR) Family Functioning (FAM-111)	11
Emotion/Person	Hamilton Rating Scales for Anxiety/Depression (HAM-A/HAM-D) Beck's Depression Inventory (BDI) General Self-Efficacy Scale (GSES)	51

##### Long-term vs. short-term

We classified studies as short-term if the treatment to follow-up was under 6 months (k = 111) and long-term if they were 6 months or more (k = 32). Combination treatments were more likely to be long-term than short-term (*k* = 6) indicating a longer follow-up. A total of 78% of pharmacological interventions were short-term with more than half of these (56%) lasting <12 weeks suggesting an immediacy to the expected effectiveness. In contrast, in psychosocial studies, therapeutic effect is assumed to be less immediate as they tend to address a wider range of symptoms and co-occurrences (Biederman et al., 2012). Long-term research into the impact of ADHD across the life span indicates that symptoms beyond the core symptoms such as functionality and anxiety become more prominent over time, yet current research does not reflect this because the majority of studies are short term and evaluating pharmacological interventions (Ingram et al., 1999).

##### Qualitative analysis

There were two studies that evaluated the effectiveness of interventions using qualitative methods (Nordby et al., 2021) with one using both qualitative and quantitative data to support their findings (Björk et al., 2020). Both studies interviewed participants about their experience of completing the interventions and extracted themes using thematic and content analysis. Their analyses support the key mechanisms identified for effective psychosocial interventions with one theme highlighting the importance of trusting relationships with the clinician delivering the intervention (Björk et al., 2020), and the other emphasizing the sense of belonging and shared experience in group interventions (Nordby et al., 2021).

#### The workplace

Our review aimed to include intervention studies that were specifically related to the workplace, carried out in workplace contexts, or involving workplace outcomes. Unfortunately, no studies were conducted in workplace contexts apart from two studies that were carried out in a simulated workplace environment (Wigal et al., 2010, p. 20). A total of four studies included workplace outcomes as one of their primary outcomes with a further 11 including a including work related outcomes as a secondary outcome.

The four studies that primarily assessed work-related outcomes were categorized as pharmacological or combined. The studies that assessed the efficacy of both pharmacological and psychosocial interventions combined had a positive impact on work outcomes improving functioning at work (Dittner et al., 2018) and maintaining employment (LaLonde et al., 2013). The pharmacological studies did not have a positive effect with there being no difference in work productivity (Adler et al., 2008) and no improvement in occupational status at follow up (Torgersen et al., 2014). Hence, the psychosocial aspect of the intervention might be a key mechanism having a direct impact on work outcomes.

We then manually searched each study to identify any outcomes relevant to the workplace but not explicitly measuring workplace performance or employment, 35 studies included measurements of outcomes that involved subscales assessing work-relevant outputs. We further grouped these instruments into two categories, those that assess general organization and time management and others that assess general life functioning, including a subscale measuring workplace functioning. General organization and time management scales were present in 13 studies and included scales like “ON-TOP” or “On Time Management Organization and Planning scale” and an adult adapted version of the Child Organisation Skills Measure (COSM). A total of eight studies used the same functioning measure entitled the Sheehan Disability Scale that requires participants to rate on a Likert scale how much they feel their disability impacts their work, family/home life and social/leisure activities. Other measures relating to life satisfaction and functioning use a similar form of including work as a domain that could be impacted. Most of the work-relevant measures lacked sufficient reliability or validity estimates or consisted of only one item which further limits the reliability of the study's findings and implications.

### Assessment of risk of bias

After the full texts were extracted, we assessed them for risk of bias (Higgins and Green, 2011). We assessed quality using a checklist of 18 questions adapted from three existing quality assessment tools recommended in the Cochrane guidance to calculate a numeric score. We drew on The Newcastle-Ottawa scale (Wells et al., 1999), the Cochrane Collaboration's tool for assessing risk of bias (Higgins and Green, 2011), and the Qualitative Research Checklist from the Critical Appraisal Skills Programme (Tong et al., 2007) and mapped their criteria on the CIMO framework to reflect the purpose and interests of the present review, see Table 6 for domains and example items. We calculated a score for each study based on the checklist which we then compared to the scores in Table 7 as an overall assessment of bias.

**Table 6:** Example items from the risk of bias tool.

**Domain**	**Example items**	**Total items**
Detection	Were participants blind to the outcome assessment?	1
Attrition	Did the data sufficiently support the findings?	2
Reporting	Have ethical issues been considered?	6
Selection	Was the recruitment strategy appropriate to the aims of the research?	4
Performance	Were participants blind to the intervention rationale?	3
Other	What was the length of follow-up in months?	3

**Table 7:** Adapted risk of bias scoring tool.

**Score /24**	**Risk of bias**	**Interpretation**
17–24	Low risk of bias	Bias, if present, is unlikely to alter the results seriously
9–16	Unclear risk of bias	A risk of bias that raises some doubt about the results
0–8	High risk of bias	Bias may alter the results seriously

Adapted from Higgins et al. (2011).

**Figure 4:**
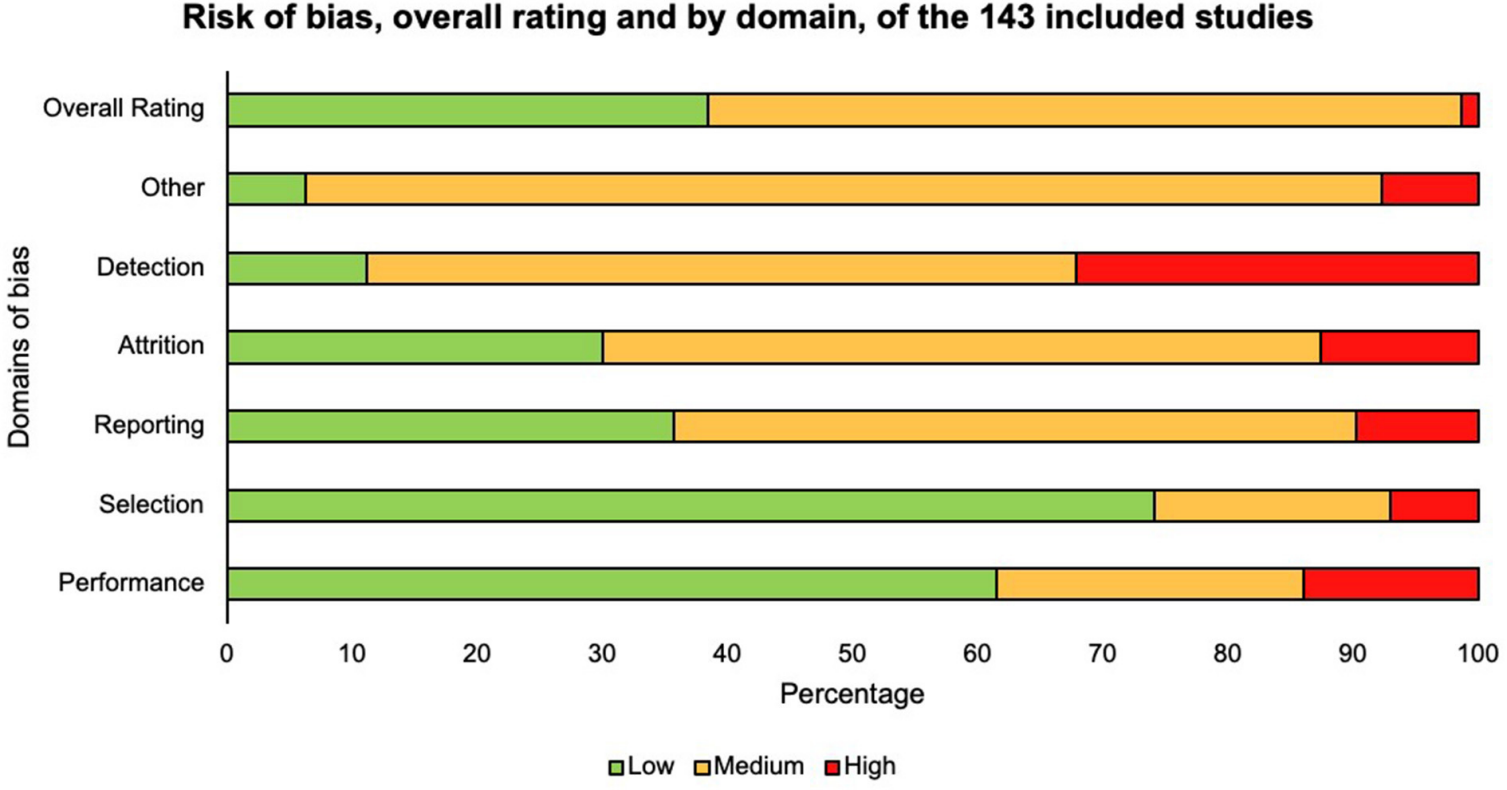
Risk of bias.

We rated most studies (60%) as unclear regarding risk of bias, which raises some doubt about the results, due to the ambiguity and the lack of detail provided in the studies' methodology and findings (see Figure 4). Regarding any psychosocial studies, it was a challenge to comprehend the details of intervention the “skills training” interventions listed the skills they targeted but did not provide examples of how these skills were targeted. The lack of detail may be due to publication restrictions on word count, privacy (training designed for commercial implications), or little theoretical application which we note upfront as a limitation regarding the generalisability of the findings. We rated a total of 1% of the studies as high risk of bias and these tended to include poorer ratings across the categories. On the other hand, a large percentage of studies were rated at a low risk of bias in domains of performance (62%) and selection (75%) indicating a strength in research around blinding of the control and the intervention group. A further strength we identified was the appropriate recruitment strategy to encourage participation from individuals who are generalisable to the ADHD population compared to recruiting student samples which are often critiqued for their lack of ecological validity (Bello et al., 2014).

## Discussion

### Summary of main findings

Of 143 studies which documented interventions to support ADHDers a large proportion has been conducted in the clinical and medical fields on the efficacy of pharmacological interventions, subsequently influencing existing policy and guidelines. Our review did not locate relevant interventions in workplace contexts, designed specifically for workplaces, based on theory relevant to the workplace, and limited research measured outcomes specific to employment. This lack of evidence signposts a clear need for robust research investigating psychosocial interventions combined with or compared to pharmacological interventions. Therefore, it is evident from the existing synthesis to build an evidence base that is transferable to the workplace. Key mechanisms to further examine and include in future research are group interventions, inclusion of the support network around the ADHDer, the clinician-patient relationship, psychoeducation, and student/patient/coachee led interventions. Important considerations for future research and practice that are based on the review findings are listed in Table 8.

**Table 8:** Findings from the realist evaluation summarized into important factors.

**Important factors in interventions for ADHDers**		
Context	Societal	Access to diagnosis and support, socioeconomic status, national and international policy and guidelines
	Settings	Applicable to a range including: Medical/educational/**workplace**
	Interpersonal	Involving others in the intervention, promoting successful clinician-patient/coach-coachee relationships
	Individual	Address co-occurrence
Intervention	Pharmacological	Blinded experimenter and both control and treatment group
	Psychosocial	Autonomy in topics/skills to address, clear methodology and detail of what the intervention involved
	Group/individual	Benefits of the group on shared experiences and meaningfulness
	Combination	Need more studies involving a combination of pharmacological and psychosocial interventions.
Mechanisms	Pharmacological Psychological	Brain chemistry Cognition and psychoeducation
Outcomes	Core symptoms	Measured by the clinician, should include participant response and family/**workplace ratings**
	Beyond core symptoms	Measure outcomes from all aspects of life and symptomatology e.g., life functioning, emotion, and anxiety.
	Long-term vs. short-term	Long-term effectiveness is imperative

Bold is for future considerations for research.

More specifically, our review findings highlighted that psychosocial interventions, especially training and coaching need to explicitly outline their methods, mechanisms, and theoretical grounding. Psychoeducation is a potential mechanism in interventions that greatly influences the efficacy and ultimately the self-awareness and understanding. Student/patient/coachee led interventions also seem to increase the effectiveness by encouraging autonomy of the challenges to address and outcomes relating to self-esteem and self-efficacy. In addition, interventions involving a significant other seem to be effective in supporting the person as a whole and increasing the knowledge of those in the individual's social network. In sum, these theoretical underpinnings can be used to guide further research.

Our review findings highlight the necessity for future intervention research aimed at supporting ADHDers to include a workplace component and assess primary work-relevant outcomes using reliable and valid scales. Intervention research should assess the efficacy of the intervention on a range of outcomes including the three core symptoms as well as skills-related outcomes and functioning in life and at work. In addition, studies should examine whether skills-based and cognitive behavioral therapies are applicable across contexts and demonstrate far transfer to the workplace. Based on the NICE guidelines, the first step in managing ADHD is to make modifications to the environment which is in line with the legal requirements for employers to make reasonable adjustments (changes to the environment) for employees with a disability. Future research and practice need to examine what adjustments and modifications are effective and revisit whether any mechanisms of existing psychosocial interventions can be applied to the workplace because this review identifies they are effective for work-relevant outcomes.

The expert panel identified disclosure of ADHD to be a significant barrier to accessing workplace support as well as access to coaching. Members of the panel highlighted the importance of psychoeducation and self-awareness of an ADHDers experiences in knowing which support is most effective for them, emphasizing a personalized approach. Therefore, structural barriers to accessing support for ADHDers should also be considered in future research and practice.

### Limitations

A limitation of the review, and many others in management research, is that it did not include any gray literature despite efforts to ask the expert panel for recommendations (Rojon et al., 2021). Gray literature is important because it can reduce publication bias and influence the review synthesis (Gough et al., 2012). Hence, there may be existing work-related interventions in practice that are or are not documented in the gray literature and these could include effective mechanisms/designs that influence the review's practical recommendations. For example, the expert panel findings indicated that coaching is often used in practice yet only two studies evaluated its efficacy (Stevenson et al., 2003; Kubik, 2010). Given the typical primary settings, we also surmise that study participants were likely to exhibit symptoms which may have inhibited workplace performance and progression. In other words, we cannot preclude that the samples may have been less likely to comprise ADHDers for example in senior, managerial or professional roles.

## Conclusions

From the 143 studies synthesized, none evaluated the efficacy of workplace interventions. Despite the lack of workplace specific intervention studies, the synthesis identifies effective mechanisms that could be applied to the workplace context such as group-based interventions and those which involve elements of psychoeducation, where the social support network around the ADHDer is included in the intervention. Interventions categorized as psychosocial were applicable to the workplace because they are likely to improve symptoms related to emotional regulation and social interactions that are important for work contexts. Workplace outcomes are often considered as secondary, and any skills-based interventions target general skills rather than specific work-relevant skills training. As a result, it is unclear whether the recommended support for ADHDers at work is evidence-based and accessible for practitioners who advise employees with ADHD and the employees themselves. There is a clear need to implement theory-driven, rigorously designed and context relevant interventions to facilitate the transfer of learning to the workplace. Furthermore, additional research is required regarding psychosocial interventions to overcome contemporary workplace challenges such as managing concurrent work tasks and navigating complex teams. Only once we address such important topics can we be confident that we have a rigorous evidence base to support ADHDers at work.

## Data availability statement

The raw data supporting the conclusions of this article will be made available by the authors, without undue reservation.

## Author contributions

KL, AM, and HT contributed to conception and design of the study. KL organized the expert panel, ran the searches, screened the studies, extracted the data, ran risk of bias assessment, and wrote the first draft of the manuscript. All authors contributed to manuscript revision, read, and approved the submitted version.

## Funding

We acknowledge funding through the Birkbeck Wellcome Trust Institutional Strategic Support Fund, award 204770/Z/16/Z, which supported a 3 months period for the writing up and refinement of the current review.

## Conflict of interest

The authors declare that the research was conducted in the absence of any commercial or financial relationships that could be construed as a potential conflict of interest.

## Publisher's note

All claims expressed in this article are solely those of the authors and do not necessarily represent those of their affiliated organizations, or those of the publisher, the editors and the reviewers. Any product that may be evaluated in this article, or claim that may be made by its manufacturer, is not guaranteed or endorsed by the publisher.
