# Supplementary material for: A systematic review of interventions to support adults with ADHD at work—Implications from the paucity of context-specific research for theory and practice
Source: Front Psychol. 2022 Aug 22;13:893469. doi: 10.3389/fpsyg.2022.893469 (PMC9443814; doi:10.3389/fpsyg.2022.893469)
Supplement: Supplementary file 1 [file Table_1.docx]

Supplementary Material

**Supplementary Table 1**

*Characteristics of included studies in final review: by author, year, intervention type, country, sample, gender, design, and follow-up.*

| **no** | **Author** | **Year** | **Intervention type** | **Country** | **Sample** | **Gender (% male)** | **Design** | **Follow-up (weeks)** |
| --- | --- | --- | --- | --- | --- | --- | --- | --- |
| 1 | Adler et al. | 2008b | Pharma | USA | 410 | 56-63 | RCT | 24 |
| 2 | Adler et al. | 2008a | Pharma | USA | 420 | 37-67 | RCT | 4 |
| 3 | Adler et al. | 2008c | Pharma | USA | 536 | 64 | SBA | 192 |
| 4 | Adler et al. | 2009a | Pharma | USA | 264 | 53.6 | RCT | 14 |
| 5 | Adler et al. | 2009c | Pharma | USA | 226 | 55-57 | RCT | 7 |
| 6 | Adler et al. | 2009b | Pharma | USA | 206 | 50 | RCT | 24 |
| 7 | Adler et al. | 2013 | Pharma | USA | 40 | 63 | SBA | 12 |
| 8 | Adler et al. | 2014 | Pharma | USA | 24 | ns | RCT | 14 |
| 9 | Adler et al. | 2020 | Pharma | USA | 502 | 53 | RCT | 52 |
| 10 | Adler et al. | 2021 | Pharma | USA | 1166 | 50 | RCT | 8 |
| 11 | Agay et al. | 2010 | Pharma | Israel | 58 | 53 | RCT | 0 |
| 12 | Amiri et al. | 2012 | Pharma | Iran | 21 | 62 | RCT | 6 |
| 13 | Anastopoulos et al. | 2021 | Psycho | USA | 250 | 33-36 | RCT | 52 |
| 14 | Apostol et al. | 2012 | Pharma | USA | 221 | 68 | RCT | 4 |
| 15 | Arnold et al. | 2014 | Pharma | USA | 330 | 53-70 | RCT | 9 |
| 16 | Bachmann et al. | 2018 | Psycho | Germany | 74 | 55 | RCT | 8 |
| 17 | Biederman et al. | 2005 | Pharma | USA | 221 | 56-66 | RCT | 104 |
| 18 | Biederman et al. | 2006 | Pharma | USA | 141 | 47-57 | RCT | 6 |
| 19 | Biederman et al. | 2007 | Pharma | USA | 285 | 50-59 | RCT | 6 |
| 20 | Biederman et al. | 2011 | Pharma | USA | 223 | 57 | RCT | 4 |
| 21 | Biederman et al. | 2019 | Pharma | USA | 344 | 48-56 | RCT | 6 |
| 22 | Björk et al. | 2020 | Psycho | Sweden | 15 | 66 | SBA | 52 |
| 23 | Bloch et al. | 2017 | Pharma | Israel | 61 | 61 | CBA | 0.14 |
| 24 | Boonstra et al. | 2005 | Pharma | Netherlands | 45 | 49 | RCT | 3 |
| 25 | Bouffard et al. | 2003 | Pharma | Canada | 42 | 80 | RCT | 2 |
| 26 | Bramham et al. | 2009 | Psycho | UK | 61 | 65 | CBA | 12 |
| 27 | Bron et al. | 2014 | Pharma | Netherlands | 22 | 88 | RCT | 6 |
| 28 | Brown et al. | 2011 | Pharma | USA | 501 | 50 | RCT | 26 |
| 29 | Bueno et al. | 2015 | Psycho | Brazil | 29 | 50 | CBA | 8 |
| 30 | Buitelaar et al. | 2012 | Pharma | Europe | 200 | 31-54 | RCT | 52 |
| 31 | Casas et al. | 2013 | Pharma | Europe | 279 | 44-52 | RCT | 13 |
| 32 | Cherkasova et al. | 2016 | Psycho | Canada | 88 | 69 | RCT | 12 |
| 33 | Cole et al. | 2016 | Psycho | Switzerland | 29 | 54 | CBA | 52 |
| 34 | Cooper et al. | 2017 | Pharma | UK | 30 | 50 | RCT | 6 |
| 35 | Corbisiero et al. | 2019 | Comb | Switzerland | 43 | 55.8 | RCT | 36 |
| 36 | Dentz et al. | 2020 | Psycho | France | 55 |  | RCT | 6 |
| 37 | Dittner et al. | 2018 | Psycho | UK | 60 | 60-79 | RCT | 15 |
| 38 | Dorrego et al. | 2002 | Pharma | Spain | 32 | 83 | RCT | 18 |
| 39 | Dotare et al. | 2020 | Psycho | Switzerland | 114 | 56 | RCT | 4 |
| 40 | Durell et al. | 2010 | Pharma | USA | 536 | 63-83 | RCT | 10 |
| 41 | Durell et al. | 2013 | Pharma | USA & Puerto Rico | 245 | 56-58 | RCT | 12 |
| 43 | Eddy et al. | 2015 | Psycho | USA | 4 | 75 | SBA | 8 |
| 44 | Eddy et al. | 2021 | Psycho | USA | 250 | 44 | RCT | 8 |
| 45 | Edel et al. | 2009 | Pharma | Germany | 24 | 38 | SBA | 20 |
| 46 | Edel et al. | 2017 | Psycho | Germany | 91 | 60 | CBA | 13 |
| 47 | Emilsson et al. | 2011 | Psycho | Iceland | 54 | 37 | RCT | 12 |
| 48 | Fan et al. | 2017 | Pharma | Taiwan | 36 | 42 | RCT | 8 |
| 49 | Faraone et al. | 2012 | Pharma | USA | 349 | ns | RCT | 4 |
| 50 | Fredriksen et al. | 2014 | Pharma | Norway | 232 | 48 | SBA | 52 |
| 51 | Frick et al. | 2020 | Pharma | USA | 411 | 51-65 | RCT | 6 |
| 52 | Fuermaier et al. | 2014 | Psycho | Netherlands | 100 | 48 | CBA | 2 |
| 53 | Fuermaier et al. | 2017 | Pharma | Germany | 103 | 49 | CBA | 0.14 |
| 54 | Gabriel | 2010 | Pharma | Canada | 32 | 75 | SBA | 12 |
| 55 | Gaur & Pallanti | 2020 | Comb | USA | 30 | 64 | CBA | 24 |
| 56 | Goodman et al. | 2017 | Pharma | USA | 279 | 50-54 | RCT | 6 |
| 57 | Goto et al. | 2017 | Pharma | Asia | 386 | 51-53 | RCT | 10 |
| 58 | Gu et al. | 2018 | Psycho | China | 56 | 53-57 | RCT | 6 |
| 59 | Hamedi et al. | 2014 | Pharma | Iran | 42 | 64 | RCT | 6 |
| 60 | Hartung et al. | 2020 | Psycho | USA | 30 | 57 | SBA | 6 |
| 61 | Hepark et al. | 2019 | Psycho | Netherlands | 103 | 54 | RCT | 12 |
| 62 | Hirvikoski et al. | 2011 | Psycho | Sweden | 51 | 26-48 | RCT | 12 |
| 63 | Hirvikoski et al. | 2017 | Psycho | Sweden | 76 | 36-47 | RCT | 8 |
| 64 | Hornig-Rohan & Amsterdam | 2002 | Pharma | USA | 17 | 71 | SBA | 6 |
| 65 | Horrigan & Barnhill | 2000 | Pharma | USA | 24 | 50 | SBA | 52 |
| 66 | Hoxhaj et al. | 2018 | Psycho | Germany | 81 | 44-53 | RCT | 8 |
| 67 | Huang et al. | 2019 | Psycho | China | 108 | 54-69 | RCT | 24 |
| 68 | Huss et al. | 2014 | Pharma | 9 countries | 725 | 51-57 | RCT | 9 |
| 42 | In der Braek et al. | 2012 | Psycho | Netherlands | 27 | 63 | RCT | 24 |
| 69 | Iwanami et al. | 2020 | Pharma | Japan | 201 | 63-66 | RCT | 10 |
| 70 | Janssen et al. | 2019 | Comb | Netherlands | 120 | 47 | RCT | 24 |
| 71 | Johnson et al. | 2010 | Pharma | Sweden | 10 | 60 | SBA | 10 |
| 72 | Kahn | 2008 | Pharma | USA | 31 | 68 | CBA | 0 |
| 73 | Koblan et al. | 2015 | Pharma | USA | 341 | 56-60 | RCT | 4 |
| 74 | Kolodny et al. | 2017 | Comb | Israel | 30 | 36 | RCT | 8 |
| 75 | Kooij et al. | 2004 | Pharma | Netherlands | 45 | 53 | RCT | 7 |
| 76 | Kubik | 2010 | Psycho | USA | 45 | 33 | CBA | 6 |
| 77 | Kuperman et al. | 2001 | Pharma | USA | 37 | 64-75 | RCT | 8 |
| 78 | LaCount et al. | 2015 | Psycho | USA | 17 | 41 | SBA | 10 |
| 79 | LaLonde et al. | 2013 | Comb | USA | 1 | 100 | SBA | 104 |
| 80 | Lee et al. | 2014 | Pharma | Asia | 74 | 35-41 | CBA | 10 |
| 81 | Lücke et al. | 2021 | Comb | Germany | 419 | 43-54 | RCT | 52 |
| 82 | Manor et al. | 2012 | Pharma | Israel | 120 | 57-65 | RCT | 6 |
| 83 | Marchant et al. | 2010 | Pharma | USA | 34 | 60-75 | RCT | 4 |
| 84 | Marchant et al. | 2011 | Pharma | USA | 52 | 61-81 | RCT | 8 |
| 85 | Mattos et al. | 2013 | Pharma | Brazil | 60 | 66.7 | SBA | 12 |
| 86 | Medori et al. | 2008 | Pharma | Europe | 304 | 45-61 | RCT | 5 |
| 87 | Mitchell et al. | 2008 | Psycho | USA | 2 | 100 | SBA | 10 |
| 88 | Ni et al. | 2017 | Pharma | Taiwan | 63 | 58-59 | RCT | 10 |
| 89 | Nicastro et al. | 2021 | Psycho | Switzerland | 38 | 50 | SBA | 8 |
| 90 | Nordby et al. | 2021 | Psycho | Norway | 10 | 70 | SBA | 9 |
| 91 | Pan et al. | 2019 | Comb | China | 124 | 53-68 | CBA | 12 |
| 92 | Pan et al. | 2021 | Psycho | China | 98 | 55-63 | RCT | 12 |
| 93 | Pettersson et al. | 2017 | Psycho | Sweden | 45 | 36 | RCT | 24 |
| 94 | Philipsen et al. | 2007 | Psycho | Germany | 72 | 60 | SBA | 13 |
| 95 | Philipsen et al. | 2015 | Comb | Germany | 419 | 43-54 | RCT | 52 |
| 96 | Puente & Mitchell | 2016 | Psycho | USA | 1 | 100 | SBA | 12 |
| 97 | Reimherr et al. | 2005 | Pharma | USA | 59 | 86 | CBA | 6 |
| 112 | Retz et al. | 2012 | Pharma | Germany | 162 | 38-56 | RCT | 8 |
| 99 | Riahi et al. | 2010 | Pharma | Iran | 86 | 45 | RCT | 6 |
| 100 | Rivkin et al. | 2012 | Pharma | USA | 36 | 58-66 | RCT | 4 |
| 101 | Rosenfield et al. | 2008 | Comb | USA | 1 | 100 | SBA | 53 |
| 102 | Rösler et al. | 2009 | Pharma | Germany | 359 | 50 | RCT | 24 |
| 103 | Rostain & Ramsay | 2006 | Comb | USA | 43 | 74 | SBA | 24 |
| 104 | Rucklidge et al. | 2014 | Psycho | New Zealand | 28 | 61 | CBA | 8 |
| 105 | Safren et al. | 2005 | Comb | USA | 31 | ns | RCT | 15 |
| 106 | Safren et al. | 2010 | Comb | USA | 86 | 55 | RCT | 12 |
| 107 | Salomone et al. | 2015 | Psycho | Ireland | 29 | 71 | RCT | 5 |
| 108 | Sobanski et al. | 2012 | Pharma | Germany | 64 | 24-60 | RCT | 12 |
| 109 | Solanto et al. | 2008 | Psycho | USA | 38 | 60 | SBA | 12 |
| 110 | Spencer et al. | 1998 | Pharma | USA | 42 | 48 | RCT | 8 |
| 111 | Spencer et al. | 2001 | Pharma | USA | 27 | 56 | RCT | 7 |
| 112 | Spencer et al. | 2005 | Pharma | USA | 146 | 54-59 | RCT | 6 |
| 113 | Spencer et al. | 2007 | Pharma | USA | 184 | 69 | RCT | 5 |
| 114 | Spencer et al. | 2011 | Pharma | USA | 44 | 49-50 | RCT | 6 |
| 115 | Stern et al. | 2016 | Psycho | Israel | 60 | 43 | RCT | 0.14 |
| 116 | Stevenson et al. | 2002 | Psycho | Australia | 43 | 67 | RCT | 8 |
| 117 | Stevenson et al. | 2003 | Psycho | Australia | 35 | 63 | RCT | 12 |
| 118 | Surman et al. | 2019 | Pharma | USA | 44 | 36.5 | RCT | 12 |
| 119 | Takahashi et al. | 2011 | Pharma | Japan | 45 | 42 | SBA | 8 |
| 120 | Takahashi et al. | 2014 | Pharma | Japan | 269 | 69-71 | RCT | 8 |
| 121 | Takahashi et al. | 2014 | Pharma | Asia | 43 | 68 | SBA | 8 |
| 122 | Torgersen et al. | 2014 | Pharma | Norway | 117 | 72 | SBA | 54 |
| 123 | Upadhyaya et al. | 2013 | Pharma | International | 349 | 57-61 | RCT | 25 |
| 124 | Virta et al. | 2008 | Psycho | Finland | 29 | 48 | SBA | 11 |
| 125 | Weisler et al. | 2006 | Pharma | USA | 255 | 29-41 | RCT | 4 |
| 126 | Weisler et al. | 2009 | Pharma | USA | 349 | 54 | SBA | 4 |
| 127 | Weisler et al. | 2017 | Pharma | USA | 275 | 47-62 | RCT | 4 |
| 128 | Weiss & Hechtman | 2006 | Comb | USA & Canada | 98 | 64 | RCT | 20 |
| 129 | Weiss et al. | 2010 | Pharma | USA & Canada | 725 | 49 | SBA | 32 |
| 130 | Weiss et al. | 2012 | Comb | USA & Canada | 53 | 65-80 | RCT | 20 |
| 131 | Weiss et al. | 2021 | Pharma | USA & Canada | 375 | 40-52 | RCT | 4 |
| 132 | Wender et al. | 2010 | Pharma | USA | 57 | 72 | SBA | 53 |
| 132 | White & Shah | 2006 | Psycho | USA | 34 | 48 | RCT | 1 |
| 134 | Wietecha et al. | 2012 | Pharma | USA | 502 | 43-51 | RCT | 24 |
| 135 | Wigal et al. | 2010 | Pharma | USA | 127 | 57-64 | RCT | 7 |
| 136 | Wigal et al. | 2018 | Pharma | USA | 85 | 41-42 | RCT | 6 |
| 137 | Wigal et al. | 2020 | Pharma | USA | 59 | 35 | RCT | 0 |
| 138 | Wiggins et al. | 1999 | Psycho | USA | 17 | 89 | CBA | 4 |
| 139 | Wilens et al. | 1996 | Pharma | USA | 41 | 51 | RCT | 6 |
| 140 | Wilens et al. | 2005 | Pharma | USA | 162 | 60 | RCT | 8 |
| 141 | Wilens et al. | 2008 | Pharma | USA | 126 | 66-74 | RCT | 8 |
| 142 | Young et al. | 2011 | Pharma | USA | 502 | 43-51 | RCT | 24 |
| 143 | Young et al. | 2015 | Comb | Iceland | 95 | 31-43 | RCT | 12 |
| *Note*. not specified (ns), pharmacological (pharma), psychosocial (psycho), combined (comb), randomised control trail (RCT), simple before and after (SBA), controlled before and after (CBA). | | | | | | | | |

**References**

References included in the systematic review are marked with an asterisk*

*Adler, L. A., Alperin, S., Leon, T., & Faraone, S. (2014). Clinical effects of lisdexamfetamine and mixed amphetamine salts immediate release in adult ADHD: results of a crossover design clinical trial. *Postgraduate medicine*, *126*(5), 17-24. <https://doi.org/10.3810/pgm.2014.09.2796>

*Adler, L. A., Frick, G., & Yan, B. (2020). A long-term, open-label, safety study of triple- bead mixed amphetamine salts (SHP465) in adults with ADHD. *Journal of Attention Disorders*, *24*(3), 434-446. [https://doi.org/10.1177/1087054717696770](https://doi.org/10.1177%2F1087054717696770)

*Adler, L. A., Goldman, R., Hopkins, S. C., Koblan, K. S., Kent, J., Hsu, J., & Loebel, A. (2021). Dasotraline in adults with attention deficit hyperactivity disorder: A placebo- controlled, fixed-dose trial. *International Clinical Psychopharmacology*, 117–125. <https://doi.org/10.1097/YIC.0000000000000333>

*Adler, L., Goodman, D. W., Kollins, S. H., Weisler, R. H., Krishnan, S., Zhang, Y., & Biederman, J. (2008a). Double-blind, placebo-controlled study of the efficacy and safety of lisdexamfetamine dimesylate in adults with attention-deficit/hyperactivity disorder. The *Journal of Clinical Psychiatry*, *69*(9), 1364– 1373. <https://doi.org/10.4088/JCP.v69n0903>

*Adler, L. A., Liebowitz, M., Kronenberger, W., Qiao, M., Rubin, R., Hollandbeck, M., ... & Durell, T. (2009a). Atomoxetine treatment in adults with attention‐deficit/hyperactivity disorder and comorbid social anxiety disorder. *Depression and Anxiety*, *26*(3), 212- 221. [**https://doi.org/10.1002/da.20549**](https://doi.org/10.1002/da.20549)

*Adler, L. A., Lynch, L. R., Shaw, D. M., Wallace, S. P., O’Donnell, K. E., Ciranni, M. A., Briggie, A. M., & Faraone, S. V. (2013). Effectiveness and Duration of Effect of Open-Label Lisdexamfetamine Dimesylate in Adults With ADHD. *Journal of Attention Disorders*, *21*(2), 149–157. https://doi.org/10.1177/1087054713485421

*Adler, L. A., Spencer, T. J., Williams, D. W., Moore, R. J., & Michelson, D. (2008c). Long- term, open-label safety and efficacy of atomoxetine in adults with ADHD: final report of a 4- year study. *Journal of Attention Disorders*, *12*(3), 248-253. <https://doi.org/10.1177/1087054708316250>

*Adler, L. A., Spencer, T., Brown, T. E., Holdnack, J., Saylor, K., Schuh, K., Trzepacz, P. T., Williams, D. W., & Kelsey, D. (2009b). Once-daily atomoxetine for adult attention- deficit/hyperactivity disorder: A 6-month, double-blind trial. Journal of Clinical *Psychopharmacology*, *29*(1), 44–50. https://doi.org/10.1097/JCP.0b013e318192e4a0

*Adler, L. A., Zimmerman, B., Starr, H. L., Silber, S., Palumbo, J., Orman, C., & Spencer, T. (2009c). Efficacy and safety of OROS methylphenidate in adults with attention- deficit/hyperactivity disorder: A randomized, placebo-controlled, double-blind, parallel group, dose-escalation study. *Journal of Clinical Psychopharmacology*, *29*(3), 239–247. <https://doi.org/10.1097/JCP.0b013e3181a390ce>

*Agay, N., Yechiam, E., Carmel, Z., & Levkovitz, Y. (2010). Non-specific effects of methylphenidate (Ritalin) on cognitive ability and decision-making of ADHD and healthy adults. *Psychopharmacology*, *210*(4), 511-519. <https://doi.org/10.1007/s00213-> 010-1853-4

*Amiri, S., Farhang, S., Ghoreishizadeh, M. A., Malek, A., & Mohammadzadeh, S. (2012). Double-blind controlled trial of venlafaxine for treatment of adults with attention deficit/hyperactivity disorder. *Human Psychopharmacology*, *27*, 76–81. https://doi.org/10.1002/hup

*Anastopoulos, A. D., Langberg, J. M., Eddy, L. D., Silvia, P. J., & Labban, J. D. (2021). A randomized controlled trial examining CBT for college students with ADHD. J*ournal of Consulting and Clinical Psychology*, *89*(1), 21. [https://doi.org/10.1037/ccp0000553](https://psycnet.apa.org/doi/10.1037/ccp0000553)

*Apostol, G., Abi-Saab, W., Kratochvil, C. J., Adler, L. A., Robieson, W. Z., Gault, L. M., ... & Saltarelli, M. D. (2012). Efficacy and safety of the novel α4β2 neuronal nicotinic receptor partial agonist ABT-089 in adults with attention-deficit/hyperactivity disorder: a randomized, double-blind, placebo-controlled crossover study. *Psychopharmacology*, *219*(3), 715-725. <https://doi.org/10.1007/s00213-011-2393-> 2

*Arnold, V. K., Feifel, D., Earl, C. Q., Yang, R., & Adler, L. A. (2014). A 9-Week, Randomized Double-Blind, Placebo-Controlled, Parallel-Group, Dose-Finding Study to Evaluate the Efficacy and Safety of Modafinil as Treatment for Adults With ADHD. *Journal of Attention Disorders*, *18*(2), 133–144. https://doi.org/10.1177/1087054712441969

*Biederman, J., Lindsten, A., Sluth, L. B., Petersen, M. L., Ettrup, A., Eriksen, H. L. F., & Fava, M. (2019). Vortioxetine for attention deficit hyperactivity disorder in adults: a randomized, double-blind, placebo-controlled, proof-of-concept study. *Journal of Psychopharmacology*, *33*(4), 511-521. [https://doi.org/10.1177/0269881119832538](https://doi.org/10.1177%2F0269881119832538)

*Biederman, J., Mick, E., Surman, C., Doyle, R., Hammerness, P., Harpold, T., ... & Spencer, T. (2006). A randomized, placebo-controlled trial of OROS methylphenidate in adults with attention-deficit/hyperactivity disorder. *Biological Psychiatry*, *59*(9), 829- 835. <https://doi.org/10.1016/j.biopsych.2005.09.011>

*Biederman, J., Mick, E. O., Surman, C., Doyle, R., Hammerness, P., Michel, E., ... & Spencer, T. J. (2007). Comparative acute efficacy and tolerability of OROS and immediate release formulations of methylphenidate in the treatment of adults with attention- deficit/hyperactivity disorder. *BMC psychiatry*, *7*(1), 1-8. <https://doi.org/10.1186/1471-> 244X-7-49

*Biederman, J., Mick, E., Fried, R., Wilner, N., Spencer, T. J., & Faraone, S. V. (2011). Are stimulants effective in the treatment of executive function deficits? Results from a randomized double blind study of OROS-methylphenidate in adults with ADHD. *European Neuropsychopharmacology*, *21*(7), 508-515. <https://doi.org/10.1016/j.euroneuro.2010.11.005>

*Biederman, J., Spencer, T. J., Wilens, T. E., Weisler, R. H., Read, S. C., & Tulloch, S. J. (2005). Long-term safety and effectiveness of mixed amphetamine salts extended release in adults with ADHD. *CNS spectrums*, *10*(S20), 16-25. doi:10.1017/S1092852900002406

*Bloch, Y., Aviram, S., Segev, A., Nitzan, U., Levkovitz, Y., Braw, Y., & Mimouni Bloch, A. (2017). Methylphenidate reduces state anxiety during a continuous performance test that distinguishes adult ADHD patients from controls. *Journal of attention disorders*, *21*(1),46-51. <https://doi.org/10.1177/1087054712474949>

- *Boonstra, A. M., Kooij, J. S., Oosterlaan, J., Sergeant, J. A., & Buitelaar, J. K. (2005). Does methylphenidate improve inhibition and other cognitive abilities in adults with childhood- onset ADHD?. *Journal of clinical and experimental neuropsychology*, *27*(3), 278-298. <https://doi.org/10.1080/13803390490515757>

*Bouffard, R., Hechtman, L., Minde, K., & Iaboni-Kassab, F. (2003). The efficacy of 2 different dosages of methylphenidate in treating adults with attention-deficit hyperactivity disorder. *The Canadian Journal of Psychiatry*, *48*(8), 546-554. <https://doi.org/10.1177/070674370304800806>

*Bron, T. I., Bijlenga, D., Boonstra, A. M., Breuk, M., Pardoen, W. F., Beekman, A. T., & Kooij, J. S. (2014). OROS-methylphenidate efficacy on specific executive functioning deficits in adults with ADHD: a randomized, placebo-controlled cross-over study. *European Neuropsychopharmacology*, *24*(4), 519-528. <https://doi.org/10.1016/j.euroneuro.2014.01.007>

*Brown, T. E., Holdnack, J., Saylor, K., Adler, L., Spencer, T., Williams, D. W., ... & Kelsey, D. (2011). Effect of atomoxetine on executive function impairments in adults with ADHD. *Journal of attention disorders*, *15*(2), 130-138. <https://doi.org/10.1177/1087054709356165>

*Buitelaar, J. K., Trott, G. E., Hofecker, M., Waechter, S., Berwaerts, J., Dejonkheere, J., & Schäuble, B. (2012). Long-term efficacy and safety outcomes with OROS- MPH in adults with ADHD. *The The International Journal of Neuropsychopharmacology*, *15*(1), 1-13. doi:10.1017/S1461145711001131

*Casas, M., Rösler, M., Kooij, J. S., Ginsberg, Y., Ramos-Quiroga, J. A., Heger, S., ... & Schäuble, B. (2013). Efficacy and safety of prolonged-release OROS methylphenidate in adults with attention deficit/hyperactivity disorder: a 13-week, randomized, double-blind, placebo-controlled, fixed-dose study. *The World Journal of Biological Psychiatry*. *14*(4), 268- 281<https://doi.org/10.3109/15622975.2011.600333>

*Cherkasova, M. V., French, L. R., Syer, C. A., Cousins, L., Galina, H., Ahmadi-Kashani, Y., & Hechtman, L. (2020). Efficacy of cognitive behavioral therapy with and without medication for adults with ADHD: A randomized clinical trial. *Journal of attention disorders*, *24*(6), 889- 903. [https://doi.org/10.1177/1087054716671197](https://doi.org/10.1177%2F1087054716671197)

*Cole, P., Weibel, S., Nicastro, R., Hasler, R., Dayer, A., Aubry, J. M., ... & Perroud, N. (2016). CBT/DBT skills training for adults with attention deficit hyperactivity disorder (ADHD). *Psychiatria Danubina*, *28*(1), 103-107.

*Cooper, R. E., Williams, E., Seegobin, S., Tye, C., Kuntsi, J., & Asherson, P. (2017). Cannabinoids in attention-deficit/hyperactivity disorder: A randomised-controlled trial. *European Neuropsychopharmacology*, *27*(8), 795-808. Doi: 10.1016/S0924- 977X(16)30912-9

*Corbisiero, S., Bitto, H., Newark, P., Abt-Mörstedt, B., Elsässer, M., Buchli-Kammermann, J., …& Stieglitz, R. D. (2018). A comparison of cognitive-behavioral therapy and pharmacotherapy vs. pharmacotherapy alone in adults with attention- deficit/hyperactivity disorder (ADHD)—A randomized controlled trial. *Frontiers in psychiatry*, *9*, 571. https://doi.org/10.3389/fpsyt.2018.00571

*Dentz, A., Guay, M. C., Parent, V., & Romo, L. (2020). Working memory training for adults with ADHD. *Journal of attention disorders*, *24*(6), 918-927. <https://doi.org/10.1177/1087054717723987>

*Dorrego, M. F., Canevaro, L., Kuzis, G., Sabe, L., & Starkstein, S. E. (2002). A randomized, double-blind, crossover study of methylphenidate and lithium in adults with attention- deficit/hyperactivity disorder: preliminary findings. *The Journal of neuropsychiatry and clinical neurosciences*, *14*(3), 289-295. <https://doi.org/10.1176/jnp.14.3.289>

*Dotare, M., Bader, M., Mesrobian, S. K., Asai, Y., Villa, A. E., & Lintas, A. (2020). Attention Networks in ADHD Adults after Working Memory Training with a Dual n-Back Task. *Brain Sciences*, *10*(10), 715. [**https://doi.org/10.3390/brainsci10100715**](https://doi.org/10.3390/brainsci10100715)

*Durell, T., Adler, L., Wilens, T., Paczkowski, M., & Schuh, K. (2010). Atomoxetine treatment for ADHD: younger adults compared with older adults. *Journal of attention disorders*, *13*(4), 401-406. [https://doi.org/10.1177/1087054709342203](https://doi.org/10.1177%2F1087054709342203)

*Durell, T. M., Adler, L. A., Williams, D. W., Deldar, A., McGough, J. J., Glaser, P. E., ... & Fox, B. K. (2013). Atomoxetine treatment of attention-deficit/hyperactivity disorder in young adults with assessment of functional outcomes: a randomized, double-blind, placebo-controlled clinical trial. *Journal of clinical psychopharmacology*, *33*(1), 45-54. doi: 10.1097/JCP.0b013e31827d8a23

*Eddy, L. D., Broman-Fulks, J. J., & Michael, K. D. (2015). Brief cognitive behavioral therapy for college students with ADHD: A case series report. *Cognitive and Behavioral Practice*, *22*(2), 127-140. <https://doi.org/10.1016/j.cbpra.2014.05.005>

*Eddy, L. D., Anastopoulos, A. D., Dvorsky, M. R., Silvia, P. J., Labban, J. D., & Langberg, J. M. (2021). An RCT of a CBT Intervention for Emerging Adults with ADHD Attending College: Functional Outcomes. *Journal of Clinical Child & Adolescent Psychology*, 1-14. https://doi.org/10.1080/15374416.2020.1867989

*Edel, M. A., Pfütze, E. M., Lieder, A., Assion, H. J., Ribbert, H., Juckel, G., & Brüne, M. (2009). Self concept, action control and ADHD symptoms under methylphenidate treatment in adults with ADHD. *Pharmacopsychiatry*, *42*(03), 109-113. DOI: 10.1055/s- 0028-1112130

*Emilsson, B., Gudjonsson, G., Sigurdsson, J. F., Baldursson, G., Einarsson, E., Olafsdottir, H., & Young, S. (2011). Cognitive behaviour therapy in medication-treated adults with ADHD and persistent symptoms: a randomized controlled trial. *BMC psychiatry*, *11*(1), 1-10. https://doi.org/10.1186/1471-244X-11-116

*Fan, L. Y., Chou, T. L., & Gau, S. S. F. (2017). Neural correlates of atomoxetine improving inhibitory control and visual processing in Drug‐naive adults with attention‐ deficit/hyperactivity disorder. *Human brain mapping*, *38*(10), 4850-4864. https://doi.org/10.1002/hbm.23683

*Faraone, S. V., Spencer, T. J., Kollins, S. H., Glatt, S. J., & Goodman, D. (2012). Dose response effects of lisdexamfetamine dimesylate treatment in adults with ADHD: an exploratory study. *Journal of attention disorders*, *16*(2), 118-127. https://doi.org/10.1177%2F1087054711403716

*Fredriksen, M., Dahl, A. A., Martinsen, E. W., Klungsøyr, O., Haavik, J., & Peleikis, D. E. (2014). Effectiveness of one-year pharmacological treatment of adult attention- deficit/hyperactivity disorder (ADHD): an open-label prospective study of time in treatment, dose, side-effects and comorbidity. *European Neuropsychopharmacology*, *24*(12), 1873-1884. https://doi.org/10.1016/j.euroneuro.2014.09.013

*Frick, G., Yan, B., & Adler, L. A. (2020). Triple-bead mixed amphetamine salts (SHP465) in adults with ADHD: Results of a phase 3, double-blind, randomized, forced-dose trial. *Journal of attention disorders*, *24*(3), 402-413. https://doi.org/10.1177%2F1087054717696771

*Fuermaier, A. B., Tucha, L., Koerts, J., van Heuvelen, M. J., van der Zee, E. A., Lange, K. W., & Tucha, O. (2014). Good vibrations–effects of whole body vibration on attention in healthy individuals and individuals with ADHD. *PLoS One*, *9*(2). https://doi.org/10.1371/journal.pone.0090747

*Fuermaier, A. B., Tucha, L., Koerts, J., Weisbrod, M., Lange, K. W., Aschenbrenner, S., & Tucha, O. (2017). Effects of methylphenidate on memory functions of adults with ADHD. *Applied Neuropsychology: Adult*, *24*(3), 199-211. https://doi.org/10.1080/23279095.2015.1124108

*Gabriel, A. (2010). The mixed amphetamine salt extended release (Adderall XR, Max-XR) as an adjunctive to SSRIS or SNRIS in the treatment of adult ADHD patients with comorbid partially responsive generalized anxiety: an open-label study. *ADHD Attention Deficit and Hyperactivity Disorders*, *2*(2), 87-92. doi:10.1007/s12402-010-0025-z

*Goodman, D. W., Starr, H. L., Ma, Y. W., Rostain, A. L., Ascher, S., & Armstrong, R. B. (2017). Randomized, 6-week, placebo-controlled study of treatment for adult attention- deficit/hyperactivity disorder: individualized dosing of osmotic-release oral system (OROS) methylphenidate with a goal of symptom remission. *The Journal of clinical psychiatry*, *78*(1), 105-114. https://doi.org/10.4088/JCP.15m10348

*Goto, T., Hirata, Y., Takita, Y., Trzepacz, P. T., Allen, A. J., Song, D. H., ... & Takahashi, M. (2017). Efficacy and safety of atomoxetine hydrochloride in Asian adults with ADHD: a multinational 10-week randomized double-blind placebo-controlled Asian study. *Journal of attention disorders*, *21*(2), 100-109. https://doi.org/10.1177%2F1087054713510352

*Hamedi, M., Mohammdi, M., Ghaleiha, A., Keshavarzi, Z., Jafarnia, M., Keramatfar, R., ... & Akhondzadeh, S. (2014). Bupropion in adults with attention-deficit/hyperactivity disorder: a randomized, double-blind study. *Acta Medica Iranica*, 675-680.

*Hornig-Rohan, M., & Amsterdam, J. D. (2002). Venlafaxine versus stimulant therapy in patients with dual diagnosis ADD and depression. *Progress in Neuro- Psychopharmacology and Biological Psychiatry*, *26*(3), 585-589. https://doi.org/10.1016/S0278-5846(01)00312-8

*Horrigan, J. P., & Barnhill, L. J. (2000). Low-dose amphetamine salts and adult attention- deficit/hyperactivity disorder. *Journal of Clinical Psychiatry*, *61*(6), 414-417

*Huang, F., Tang, Y. L., Zhao, M., Wang, Y., Pan, M., Wang, Y., & Qian, Q. (2019). Cognitive- Behavioral therapy for adult ADHD: a randomized clinical trial in China. *Journal of attention disorders*, *23*(9), 1035-1046. https://doi.org/10.1177%2F1087054717725874

*Huss, M., Ginsberg, Y., Tvedten, T., Arngrim, T., Philipsen, A., Carter, K., ... & Kumar, V. (2014). Methylphenidate hydrochloride modified-release in adults with attention deficit hyperactivity disorder: a randomized double-blind placebo-controlled trial. *Advances in therapy*, *31*(1), 44-65. https://doi.org/10.1007/s12325-013-0085-5

*Iwanami, A., Saito, K., Fujiwara, M., Okutsu, D., & Ichikawa, H. (2020). Safety and efficacy of guanfacine extended-release in adults with attention-deficit/hyperactivity disorder: an open- label, long-term, phase 3 extension study. *BMC psychiatry*, *20*(1), 1-12. https://doi.org/10.1186/s12888-020-02867-8

*Janssen, L., Kan, C. C., Carpentier, P. J., Sizoo, B., Hepark, S., Schellekens, M. P., ... & Speckens, A. E. (2019). Mindfulness-based cognitive therapy v. treatment as usual in adults with ADHD: a multicentre, single-blind, randomised controlled trial. *Psychological medicine*, *49*(1), 55-65. doi:10.1017/S0033291718000429

*Johnson, M., Cederlund, M., Råstam, M., Areskoug, B., & Gillberg, C. (2010). Open-label trial of atomoxetine hydrochloride in adults with ADHD. *Journal of attention disorders*, *13*(5), 539- 545. https://doi.org/10.1177%2F1087054709332372

*Koblan, K. S., Hopkins, S. C., Sarma, K., Jin, F., Goldman, R., Kollins, S. H., & Loebel, A. (2015). Dasotraline for the treatment of attention-deficit/hyperactivity disorder: a randomized, double-blind, placebo-controlled, proof-of-concept trial in adults. *Neuropsychopharmacology*, *40*(12), 2745-2752. https://doi.org/10.1038/npp.2015.124

*Kolodny, T., Ashkenazi, Y., Farhi, M., & Shalev, L. (2017). Computerized progressive attention training (CPAT) vs. active control in adults with ADHD. *Journal of Cognitive Enhancement*, *1*(4), 526-538. https://doi.org/10.1007/s41465-017-0056-x

*Kooij, J. J. S., Burger, H., Boonstra, A. M., Van der Linden, P. D., Kalma, L. E., & Buitelaar, J.K. (2004). Efficacy and safety of methylphenidate in 45 adults with attention- deficit/hyperactivity disorder. A randomized placebo-controlled double-blind cross-over trial. *Psychological medicine*, *34*(6), 973-982. doi:10.1017/S0033291703001776

*Kuperman, S., Perry, P. J., Gaffney, G. R., Lund, B. C., Bever-Stille, K. A., Arndt, S., ... & Paulsen, J. S. (2001). Bupropion SR vs. methylphenidate vs. placebo for attention deficit hyperactivity disorder in adults. *Annals of Clinical Psychiatry*, *13*(3), 129-134. https://doi.org/10.1023/A:1012239823148

*LaCount, P. A., Hartung, C. M., Shelton, C. R., Clapp, J. D., & Clapp, T. K. (2015). Preliminary evaluation of a combined group and individual treatment for college students with attention- deficit/hyperactivity disorder. *Cognitive and Behavioral Practice*, *22*(2), 152-160. https://doi.org/10.1016/j.cbpra.2014.07.004

*Lee, S. I., Song, D. H., Shin, D. W., Kim, J. H., Lee, Y. S., Hwang, J. W., ... & Treuer, T. (2014). Efficacy and safety of atomoxetine hydrochloride in Korean adults with attention‐ deficit hyperactivity disorder. *Asia‐Pacific Psychiatry*, *6*(4), 386-396. https://doi.org/10.1111/appy.12160

*Lücke, C., Jenkner, C., Graf, E., Matthies, S., Borel, P., Sobanski, E., ... & Philipsen, A. (2021). Long-term improvement of quality of life in adult ADHD-results of the randomized multimodal COMPAS trial. *International Journal of Mental Health*, 1-21. https://doi.org/10.1080/00207411.2021.1910172

*Manor, I., Ben-Hayun, R., Aharon-Peretz, J., Salomy, D., Weizman, A., Daniely, Y., ... & Adler, L. A. (2012). A randomized, double-blind, placebo-controlled, multicenter study evaluating the efficacy, safety, and tolerability of extended-release metadoxine in adults with attention-deficit/hyperactivity disorder. *The Journal of clinical psychiatry*, *73*(12). https://doi.org/10.4088/JCP.12m07767

*Marchant, B. K., Reimherr, F. W., Halls, C., Williams, E. D., & Strong, R. E. (2010). OROS methylphenidate in the treatment of adults with ADHD: a 6-month, open-label, follow-up study. *Ann Clin Psychiatry*, *22*(3), 196-204.

*Marchant, B. K., Reimherr, F. W., Robison, R. J., Olsen, J. L., & Kondo, D. G. (2011). Methylphenidate transdermal system in adult ADHD and impact on emotional and oppositional symptoms. *Journal of attention disorders*, *15*(4), 295-304. https://doi.org/10.1177%2F1087054710365986

*Mattos, P., Louzã, M. R., Palmini, A. L. F., Oliveira, I. R. D., & Rocha, F. L. (2013). A Multicenter, Open-Label Trial to Evaluate the Quality of Life in Adults with ADHD Treated with Long-Acting Methylphenidate (OROS MPH) Concerta Quality of Life (Conqol) Study. *Journal of attention disorders*, *17*(5), 444-448. https://doi.org/10.1177%2F1087054711434772

*Medori, R., Ramos-Quiroga, J. A., Casas, M., Kooij, J. J. S., Niemelä, A., Trott, G. E., ... & Buitelaar, J. K. (2008). A randomized, placebo-controlled trial of three fixed dosages of prolonged-release OROS methylphenidate in adults with attention- deficit/hyperactivity disorder. *Biological psychiatry*, *63*(10), 981-989. https://doi.org/10.1016/j.biopsych.2007.11.008

*Mitchell, J. T., Nelson-Gray, R. O., & Anastopoulos, A. D. (2008). Adapting an emerging empirically supported cognitive-behavioral therapy for adults with ADHD and comorbid complications: An example of two case studies. *Clinical Case Studies*, *7*(5), 423-448. https://doi.org/10.1177%2F1534650108316934

*Ni, H. C., Lin, Y. J., Gau, S. S. F., Huang, H. C., & Yang, L. K. (2017). An open-label, randomized trial of methylphenidate and atomoxetine treatment in adults with ADHD. *Journal of attention disorders*, *21*(1), 27-39. https://doi.org/10.1177%2F1087054713476549

*Pan, M. R., Huang, F., Zhao, M. J., Wang, Y. F., Wang, Y. F., & Qian, Q. J. (2019). A comparison of efficacy between cognitive behavioral therapy (CBT) and CBT combined with medication in adults with attention-deficit/hyperactivity disorder (ADHD). *Psychiatry research*, *279*, 23- 33. https://doi.org/10.1016/j.psychres.2019.06.040

*Pan, M. R., Zhang, S. Y., Qiu, S. W., Liu, L., Li, H. M., Zhao, M. J., ... & Qian, Q. J. (2021). Efficacy of cognitive behavioural therapy in medicated adults with attention- deficit/hyperactivity disorder in multiple dimensions: a randomised controlled trial. *European Archives of Psychiatry and Clinical Neuroscience*, 1-21. https://doi.org/10.1007/s00406-021-01236-0

*Pettersson, R., Söderström, S., Edlund-Söderström, K., & Nilsson, K. W. (2017). Internet- based cognitive behavioral therapy for adults with ADHD in outpatient psychiatric care: A randomized trial. *Journal of attention disorders*, *21*(6), 508-521. https://doi.org/10.1177%2F1087054714539998

*Philipsen, A., Jans, T., Graf, E., Matthies, S., Borel, P., Colla, M., ... & van Elst, L. T. (2015). Effects of group psychotherapy, individual counseling, methylphenidate, and placebo in the treatment of adult attention-deficit/hyperactivity disorder: a randomized clinical trial. *JAMA psychiatry*, *72*(12), 1199-1210. doi:10.1001/jamapsychiatry.2015.2146

*Puente, A. N., & Mitchell, J. T. (2016). Cognitive-behavioral therapy for adult ADHD: A case study of multi-method assessment of executive functioning in clinical practice and manualized treatment adaptation. *Clinical Case Studies*, *15*(3), 198-211. https://doi.org/10.1177%2F1534650115614098

*Reimherr, F. W., Hedges, D. W., Strong, R. E., Marchant, B. K., & Williams, E. D. (2005). Bupropion SR in adults with ADHD: a short-term, placebo-controlled trial. *Neuropsychiatric Disease And Treatment*, *1*(3), 245. PMID: 18568102; PMCID: PMC2416755.

*Retz, W., Rösler, M., Ose, C., Scherag, A., Alm, B., Philipsen, A., ... & Study Group. (2012). Multiscale assessment of treatment efficacy in adults with ADHD: a randomized placebo- controlled, multi-centre study with extended-release methylphenidate. *The World Journal of Biological Psychiatry*, *13*(1), 48-59. https://doi.org/10.3109/15622975.2010.540257

*Riahi, F., Tehrani‐Doost, M., Shahrivar, Z., & Alaghband‐Rad, J. (2010). Efficacy of reboxetine in adults with attention‐deficit/hyperactivity disorder: A randomized, placebo‐controlled clinical trial. *Human Psychopharmacology: Clinical and Experimental*, *25*(7‐8), 570-576. https://doi.org/10.1002/hup.1158

*Rivkin, A., Alexander, R. C., Knighton, J., Hutson, P. H., Wang, X. J., Snavely, D. B., ... & Adler, L. A. (2012). A randomized, double-blind, crossover comparison of MK-0929 and placebo in the treatment of adults with ADHD. *Journal of Attention Disorders*, *16*(8), 664- 674. https://doi.org/10.1177%2F1087054711423633

*Rosenfield, B. M., Ramsay, J. R., & Rostain, A. L. (2008). Extreme makeover: The case of a young adult man with severe ADHD. *Clinical Case Studies*, *7*(6), 471-490. https://doi.org/10.1177%2F1534650108319912

*Rösler, M., Fischer, R., Ammer, R., Ose, C., & Retz, W. (2009). A randomised, placebo- controlled, 24-week, study of low-dose extended-release methylphenidate in adults with attention-deficit/hyperactivity disorder. *European archives of psychiatry and clinical neuroscience*, *259*(2), 120-129. https://doi.org/10.1007/s00406-008-0845-4

*Rostain, A. L., & Ramsay, J. R. (2006). A combined treatment approach for adults with ADHD- results of an open study of 43 patients. *Journal of attention disorders*, *10*(2), 150-159. https://doi.org/10.1177%2F1087054706288110

*Rucklidge, J. J., Frampton, C. M., Gorman, B., & Boggis, A. (2014). Vitamin–mineral treatment of attention-deficit hyperactivity disorder in adults: double-blind randomised placebo-controlled trial. *The British Journal of Psychiatry*, *204*(4), 306-315. doi:10.1192/bjp.bp.113.132126

*Safren, S. A., Otto, M. W., Sprich, S., Winett, C. L., Wilens, T. E., & Biederman, J. (2005). Cognitive-behavioral therapy for ADHD in medication-treated adults with continued symptoms. *Behaviour research and therapy*, *43*(7), 831-842. https://doi.org/10.1016/j.brat.2004.07.001

*Safren, S. A., Sprich, S., Mimiaga, M. J., Surman, C., Knouse, L., Groves, M., & Otto, M. W. (2010). Cognitive behavioral therapy vs relaxation with educational support for medication-treated adults with ADHD and persistent symptoms: a randomized controlled trial. *JAMA*, *304*(8), 875-880. doi:10.1001/jama.2010.1192

*Salomone, S., Fleming, G. R., Shanahan, J. M., Castorina, M., Bramham, J., O’Connell, R.G., & Robertson, I. H. (2015). The effects of a Self-Alert Training (SAT) program in adults with ADHD. *Frontiers in human neuroscience*, *9*, 45. https://doi.org/10.3389/fnhum.2015.00045

*Sobanski, E., Sabljic, D., Alm, B., Baehr, C., Dittmann, R. W., Skopp, G., & Strohbeck- Kuehner, P. (2012). A randomized, waiting list-controlled 12-week trial of atomoxetine in adults with ADHD. *Pharmacopsychiatry*, *45*(03), 100-107. doi: 10.1055/s-0031-1291319

*Solanto, M. V., Marks, D. J., Mitchell, K. J., Wasserstein, J., & Kofman, M. D. (2008). Development of a new psychosocial treatment for adult ADHD. *Journal of Attention Disorders*, *11*(6), 728-736. https://doi.org/10.1177%2F1087054707305100

*Spencer, T., Biederman, J., Wilens, T., Prince, J., Hatch, M., Jones, J., ... & Seidman, L. (1998). Effectiveness and tolerability of tomoxetine in adults with attention deficit hyperactivity disorder. *American Journal of Psychiatry*, *155*(5), 693-695. https://doi.org/10.1176/ajp.155.5.693

*Spencer, T. J., Adler, L. A., McGough, J. J., Muniz, R., Jiang, H., Pestreich, L., & Adult ADHD Research Group. (2007). Efficacy and safety of dexmethylphenidate extended-release capsules in adults with attention-deficit/hyperactivity disorder. *Biological Psychiatry*, *61*(12), 1380-1387. https://doi.org/10.1016/j.biopsych.2006.07.032

*Spencer, T., Biederman, J., Wilens, T., Doyle, R., Surman, C., Prince, J., ... & Faraone, S. (2005). A large, double-blind, randomized clinical trial of methylphenidate in the treatment of adults with attention-deficit/hyperactivity disorder. *Biological psychiatry*, *57*(5), 456- 463. https://doi.org/10.1016/j.biopsych.2004.11.043

*Spencer, T., Biederman, J., Wilens, T., Faraone, S., Prince, J., Gerard, K., ... & Bearman, S.K. (2001). Efficacy of a mixed amphetamine salts compound in adults with attention- deficit/hyperactivity disorder. *Archives of general psychiatry*, *58*(8), 775-782. doi:10.1001/archpsyc.58.8.775

*Spencer, T., Mick, E., Surman, C. B., Hammerness, P., Doyle, R., Aleardi, M., ... & Biederman, J. (2011). A randomized, single-blind, substitution study of OROS methylphenidate (Concerta) in ADHD adults receiving immediate release methylphenidate. *Journal of attention disorders*, *15*(4), 286-294. https://doi.org/10.1177%2F1087054710367880

*Stern, A., Malik, E., Pollak, Y., Bonne, O., & Maeir, A. (2016). The efficacy of computerized cognitive training in adults with ADHD: A randomized controlled trial. *Journal of attention disorders*, *20*(12), 991-1003. https://doi.org/10.1177%2F1087054714529815

*Surman, C., Ceranoglu, A., Vaudreuil, C., Albright, B., Uchida, M., Yule, A., ... & Biederman, J. (2019). Does L-methylfolate supplement methylphenidate pharmacotherapy in attention- deficit/hyperactivity disorder?: Evidence of lack of benefit from a double-blind, placebo- controlled, randomized clinical trial. *Journal of clinical psychopharmacology*, *39*(1), 28. doi: 10.1097/JCP.0000000000000990

*Takahashi, M., Goto, T., Takita, Y., Chung, S. K., Wang, Y., & Gau, S. S. F. (2014). Open‐ label, dose‐titration tolerability study of atomoxetine hydrochloride in Korean, Chinese, and Taiwanese adults with attention‐deficit/hyperactivity disorder. *Asia‐Pacific Psychiatry*, *6*(1), 62-70. https://doi.org/10.1111/j.1758-5872.2012.00204.x

*Takahashi, M., Takita, Y., Goto, T., Ichikawa, H., Saito, K., Matsumoto, H., & Tanaka, Y. (2011). An open‐label, dose‐titration tolerability study of atomoxetine hydrochloride in Japanese adults with attention‐deficit/hyperactivity disorder. *Psychiatry and clinical neurosciences*, *65*(1), 55-63. https://doi.org/10.1111/j.1440-1819.2010.02159.x

*Takahashi, N., Koh, T., Tominaga, Y., Saito, Y., Kashimoto, Y., & Matsumura, T. (2014). A randomized, double-blind, placebo-controlled, parallel-group study to evaluate the efficacy and safety of osmotic-controlled release oral delivery system methylphenidate HCl in adults with attention-deficit/hyperactivity disorder in Japan. *The World Journal of Biological Psychiatry*, *15*(6), 488-498. https://doi.org/10.3109/15622975.2013.868925

*Upadhyaya, H., Ramos-Quiroga, J. A., Adler, L. A., Williams, D., Tanaka, Y., Lane, J. R., ... & Allen, A. J. (2013). Maintenance of response after open-label treatment with atomoxetine hydrochloride in international European and non-European adult outpatients with attention- deficit/hyperactivity disorder: a placebo-controlled, randomised withdrawal study. *The European Journal of Psychiatry*, *27*(3), 185-205. <https://dx.doi.org/10.4321/S0213-> 61632013000300004

*Weisler, R. H., Biederman, J., Spencer, T. J., Wilens, T. E., Faraone, S. V., Chrisman, A. K., ... & Tulloch, S. J. (2006). Mixed amphetamine salts extended-release in the treatment of adult ADHD: a randomized, controlled trial. *CNS Spectrums*, *11*(8), 625-639. https://doi.org/10.1017/S1092852900013687

*Weisler, R., Young, J., Mattingly, G., Gao, J., Squires, L., & Adler, L. (2009). Long-term safety and effectiveness of lisdexamfetamine dimesylate in adults with attention- deficit/hyperactivity disorder. *CNS spectrums*, *14*(10), 573-586. doi:10.1017/S1092852900024056

*Weisler, R. H., Greenbaum, M., Arnold, V., Yu, M., Yan, B., Jaffee, M., & Robertson, B. (2017). Efficacy and safety of SHP465 mixed amphetamine salts in the treatment of attention-deficit/hyperactivity disorder in adults: results of a randomized, double-blind, placebo-controlled, forced-dose clinical study. *CNS drugs*, *31*(8), 685-697. https://doi.org/10.1007/s40263-017-0455-7

*Weiss, M., Hechtman, L., & Adult ADHD Research Group. (2006). A randomized double- blind trial of paroxetine and/or dextroamphetamine and problem-focused therapy for attention-deficit/hyperactivity disorder in adults. *The Journal of clinical psychiatry*, *67*(4), 16805.

*Weiss, M. D., Gibbins, C., Goodman, D. W., Hodgkins, P. S., Landgraf, J. M., & Faraone, S. V. (2010). Moderators and mediators of symptoms and quality of life outcomes in an open-label study of adults treated for attention-deficit/hyperactivity disorder. *The Journal of clinical psychiatry*, *71*(4), 12134. doi: 10.4088/JCP.08m04709pur

*Weiss, M., Murray, C., Wasdell, M., Greenfield, B., Giles, L., & Hechtman, L. (2012). A randomized controlled trial of CBT therapy for adults with ADHD with and without medication. *BMC psychiatry*, *12*(1), 1-8. https://doi.org/10.1186/1471-244X-12-30

*Weiss, M. D., Childress, A. C., & Donnelly, G. A. (2021). Efficacy and safety of PRC-063, extended-release multilayer methylphenidate in adults with ADHD including 6-month open-label extension. *Journal of Attention Disorders*, *25*(10), 1417-1428. https://doi.org/10.1177%2F1087054719896853

*Wender, P. H., Reimherr, F. W., Marchant, B. K., Sanford, M. E., Czajkowski, L. A., & Tomb, D.A. (2010). A one year trial of methylphenidate in the treatment of ADHD. *Journal of Attention Disorders*, *15*(1), 36-45. https://doi.org/10.1177%2F1087054709356188

*White, H. A., & Shah, P. (2006). Training attention-switching ability in adults with ADHD. *Journal of Attention Disorders*, *10*(1), 44-53. https://doi.org/10.1177%2F1087054705286063

*Wietecha, L., Young, J., Ruff, D., Dunn, D., Findling, R. L., & Saylor, K. (2012). Atomoxetine once daily for 24 weeks in adults with attention-deficit/hyperactivity disorder (ADHD): impact of treatment on family functioning. *Clinical neuropharmacology*, *35*(3), 125-133. doi: 10.1097/WNF.0b013e3182560315

*Wigal, T. L., Newcorn, J. H., Handal, N., Wigal, S. B., Mulligan, I., Schmith, V., & Konofal, E. (2018). A double-blind, placebo-controlled, phase II study to determine the efficacy, safety, tolerability and pharmacokinetics of a controlled release (CR) formulation of mazindol in adults with DSM-5 attention-deficit/hyperactivity disorder (ADHD). *CNS drugs*, *32*(3), 289- 301. https://doi.org/10.1007/s40263-018-0503-y

*Wigal, S. B., Wigal, T., Childress, A., Donnelly, G. A., & Reiz, J. L. (2020). The time course of effect of multilayer-release methylphenidate hydrochloride capsules: a randomized, double-blind study of adults with ADHD in a simulated adult workplace environment. *Journal of Attention Disorders*, *24*(3), 373-383. https://doi.org/10.1177%2F1087054716672335

*Wilens, T. E., Biederman, J., Prince, J., Spencer, T. J., Faraone, S. V., Warburton, R., ... & Geller, D. (1996). Six-week, double-blind, placebo-controlled study of desipramine for adult attention deficit hyperactivity disorder. *American Journal of Psychiatry*, *153*(9), 1147-1153.

*Wilens, T. E., Haight, B. R., Horrigan, J. P., Hudziak, J. J., Rosenthal, N. E., Connor, D. F., ... & Modell, J. G. (2005). Bupropion XL in adults with attention-deficit/hyperactivity disorder: a randomized, placebo-controlled study. *Biological psychiatry*, *57*(7), 793-801. https://doi.org/10.1016/j.biopsych.2005.01.027

*Wilens, T. E., Klint, T., Adler, L., West, S., Wesnes, K., Graff, O., & Mikkelsen, B. (2008). A randomized controlled trial of a novel mixed monoamine reuptake inhibitor in adults with ADHD. *Behavioral and Brain Functions*, *4*(1), 1-10. https://doi.org/10.1186/1744-9081-4-24

*Young, J. L., Sarkis, E., Qiao, M., & Wietecha, L. (2011). Once-daily treatment with atomoxetine in adults with attention-deficit/hyperactivity disorder: a 24-week, randomized, double-blind, placebo-controlled trial. *Clinical neuropharmacology*, *34*(2), 51-60. doi: 10.1097/WNF.0b013e31820c00eb

*Young, S., Khondoker, M., Emilsson, B., Sigurdsson, J. F., Philipp-Wiegmann, F., Baldursson, G., ... & Gudjonsson, G. (2015). Cognitive–behavioural therapy in medication-treated adults with attention-deficit/hyperactivity disorder and co-morbid psychopathology: a randomized controlled trial using multi-level analysis. *Psychological medicine*, *45*(13), 2793-2804. https://doi.org/10.1017/S0033291715000756
